# Wide-Range, Rapid, and Specific Identification of Pathogenic Bacteria by Surface-Enhanced Raman Spectroscopy

**DOI:** 10.1021/acssensors.1c00641

**Published:** 2021-07-20

**Authors:** Siying Liu, Qiushi Hu, Chao Li, Fangrong Zhang, Hongjing Gu, Xinrui Wang, Shuang Li, Lei Xue, Tobias Madl, Yun Zhang, Lei Zhou

**Affiliations:** † CAS Key Laboratory of Design and Assembly of Functional Nanostructures, and Fujian Provincial Key Laboratory of Nanomaterials, Fujian Institute of Research on the Structure of Matter, Chinese Academy of Sciences, Fuzhou 350002, China; ‡ Department of Translational Medicine, Xiamen Institute of Rare Earth Materials, Chinese Academy of Sciences, Xiamen 361021, China; § University of Chinese Academy of Sciences, Beijing 100049, China; ∥ State Key Laboratory of Biochemical Engineering, PLA Key Laboratory of Biopharmaceutical Production & Formulation Engineering, Institute of Process Engineering, Chinese Academy of Sciences, Beijing 100190, China; ⊥ Institute of Medical Equipment, Academy of Military Sciences, Tianjin 300161, China; # Gottfried Schatz Research Center for Cell Signaling, Metabolism and Aging, Institute of Molecular Biology & Biochemistry, Medical University of Graz, Neue Stiftingtalstrasse 6, 8010 Graz, Austria; ¶ BioTechMed-Graz, Graz, Austria; ∇ State Key Laboratory of Pathogen and Biosecurity, Beijing Institute of Microbiology and Epidemiology, Beijing 100071, China; ○ Anti-plague Institute Hebei Province, Zhangjiakou 075000, China

**Keywords:** AgNR substrate, bacterial diagnostics, label-free, pathogenic bacteria, receiver operating characteristic analysis, surface-enhanced Raman spectroscopy (SERS)

## Abstract

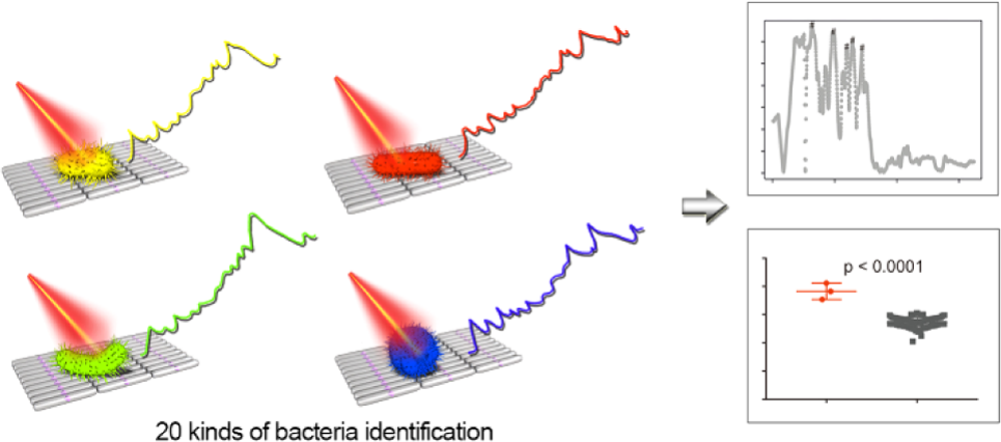

Sensitive, selective, rapid, and label-free detection of pathogenic bacteria with high generality is of great importance for clinical diagnosis, biosecurity, and public health. However, most traditional approaches, such as microbial cultures, are time-consuming and laborious. To circumvent these problems, surface-enhanced Raman spectroscopy (SERS) appears to be a powerful technique to characterize bacteria at the single-cell level. Here, by SERS, we report a strategy for the rapid and specific detection of 22 strains of common pathogenic bacteria. A novel and high-quality silver nanorod SERS substrate, prepared by the facile interface self-assembly method, was utilized to acquire the chemical fingerprint information of pathogens with improved sensitivity. We also applied the mathematical analysis methods, such as the *t*-test and receiver operating characteristic method, to determine the Raman features of these 22 strains and demonstrate the clear identification of most bacteria (20 strains) from the rest and also the reliability of this SERS sensor. This rapid and specific strategy for wide-range bacterial detection offers significant advantages over existing approaches and sets the base for automated and onsite detection of pathogenic bacteria in a complex real-life situation.

With the advanced globalization and increased interpersonal communication worldwide, pathogenic bacteria can transmit to the whole world within days. However, due to the extremely low minimum infective dose and lack of reliable characterization methods with the single-cell sensitivity,^1^ the identification and disposal of pathogenic contamination remain challenging for governments and public health organizations. Conventional biosecurity surveillance and control of high-risk areas, for example, airports and national boundaries, still rely on monitoring the post-infectious symptoms, especially human body temperature. To prevent the wide spread of pathogenic bacteria, the development of sensitive, portable, and rapid detection methods for onsite determination of pathogens is indispensable.

There have been numerous techniques to track the biological identity of pathogenic bacteria, such as chemical staining, optical microscopy, microbial culture, immunoassays, and nucleic acid identification by polymerase chain reaction.^2,3^ Nevertheless, these technologies are usually time-consuming and laborious, making them unsuitable for onsite identification.^4−9^ To this end, surface-enhanced Raman spectroscopy (SERS) emerges as a powerful tool to detect a vast array of chemical and biochemical systems.^10−18^ SERS enhancement is mainly based on the surface plasmon effect, that is, the resonant collective oscillation of conduction band electrons of certain materials, such as Au and Ag. It leads to the dramatic enhancement of the electromagnetic (EM) field in the vicinity of these nanostructures, and as a result, both the excitation and scattering light will be amplified. In the visible and near-infrared (IR) wavelength region, Ag exhibited superior plasmonic response over other noble metals.^19^ Therefore, due to high sensitivity, Ag nanostructure-based SERS substrates have been widely explored in bioanalysis. Considering the bioanalytical strategies developed for sensing biosystems, SERS can be divided into two categories, labeled (indirect) and label-free (direct) detection.^20,21^ With regard to labeled SERS detection, plasmonic nanoparticles are modified with SERS tags and targeting ligands. One can detect the SERS signal of tags when ligands attach to the targets of interest. Nonetheless, in this scenario, the advantage of SERS to directly obtain the molecular vibrational information is compromised. Therefore, by directly measuring the Raman signal of molecules of interest, label-free SERS detection offers a more straightforward method to analyze the biological systems. It is worth mentioning that, however, due to the complicated biological environment, the corresponding SERS spectra are complex, and the mathematical statistics methods are often employed to extract useful information. There have been several reports utilizing label-free SERS to verify different strains of bacteria. Corroborating with discriminant analysis, Yuan et al.^22^ used a simple SERS substrate prepared by dropping Au@Ag nanoparticles on mussel shells to distinguish Gram-negative *Escherichia coli* and *Pseudomonas aeruginosa* from Gram-positive *Salmonella aureus*. Also, Witkowska et al.^23^ combined the Ag–Au sensor with principal component analysis, achieving the differentiation of as many as nine genoserogroups of *Listeria monocytogenes*, which indicated the intrinsic variation of different genotypes for the same strain. However, the wide-range identification of pathogens (more than 20 strains) still remains unfulfilled. It is always demanding to further expand the application generality of SERS substrates.

In this study, we demonstrate a novel approach for real-time, facile microbial identification of as many as 20 bacteria strains using the SERS substrate. Specifically, a silver nanorod (AgNR)-based SERS substrate developed by our group^28,29^ was optimized for bacterial analysis. To prove the feasibility of this SERS sensor, 22 bacterial strains were investigated. Using mathematical analysis methods, unique spectral features of bacteria strains were uncovered, which allows for the clear identification of 20 bacteria from the rest with high sensitivity and specificity.

## Experimental Section

### Materials

Ethanol (99%), dichloromethane (99%), cyclohexane (99%), and octane (99%) were purchased from Sinopharm Chemical Reagent Co., Ltd. Poly(vinylpyrrolidone) (PVP, *M*_w_ (55000)), gold chloride trihydrate (HAuCl_4_·3H_2_O, 99%), and copper(II) chloride (CuCl_2_) were purchased from Sigma-Aldrich. Silver nitrate (AgNO_3_, 99%) and *N*,*N*-dimethylformamide (99%) were purchased from Beijing Chemical Industry Group Co., Ltd. Hexadecyltrimethylammonium chloride (CTAC) was purchased from Energy Chemical. Ascorbic acid was purchased from Shanghai Aladdin Biochemical Technology Co., Ltd. Bacteria used in the research include 12 species of Gram-negative bacteria (*Bacillus melitensis*, *Francisella tularensis*, *Yersinia pestis*, *E. coli* O157, *Salmonella paratyphi* A, *S. paratyphi* B, *S. paratyphi* C, *Salmonella typhi*, *Salmonella typhimurium*, *Salmonella enteritidis*, *Salmonella Choleraesuis,* and *Vibrio parahaemolyticus*), 9 species of Gram-positive bacteria (*L. monocytogenes*, *L. innocua*, *Bacillus anthracis* (spore), *Bacillus subtilis* (spore), *Bacillus thuringiensis* (spore), *Bacillus cereus* (spore), *B. subtilis* (spore), *S. aureus,* and *Cryptococcus neoformans*), and 1 species of acid-fast bacteria (*Mycobacterium smegmatis*). Therein, *B. melitensis* and *Y. pestis* were obtained from Anti-plague Institute Hebei Province, Zhangjiakou 075000, China. *F. tularensis*, *E. coli* O157, *B. anthracis,* and *B. subtilis* var. *niger* were obtained from State Key Laboratory of Pathogen and Biosecurity, Beijing Institute of Microbiology and Epidemiology, Beijing 100071, China. Other bacteria were purchased from BeNa Culture Collection, and the details can be seen in Table S1.

### Instruments

The mainly used instruments were Agilent Carry 5000, cold field-emission scanning electron microscope (JSM-6700F), and HORIBA microscopic confocal laser Raman spectrometer (LabRAM HR Evolution).

### Preparation and Characterization of AgNR Substrates

The synthesis of AgNRs was carried out by the method previously reported by us.^24,25^ In short, AgNRs were grown along the periphery of the gold species of the five-fold twin structure by reducing AgNO_3_ in the CTAC system. After purification, the ligands were exchanged to be PVP to obtain AgNRs in an ethanol solution with long-term stability. Using the air–liquid interface-assisted self-assembly technique, 200 μL of AgNR solution was mixed with 200 μL of CH_2_Cl_2_ and 200 μL of cyclohexane, and then, 30 μL of *n*-octane was added. The mixed solution was dripped on the water surface, and as the mixed solution was added, the silver film began to form, and finally, a uniform single-layer silver film was obtained. The silver film was transferred by using a clean silicon wafer and dried for subsequent detection of SERS. The AgNR substrate with multiple layers simply required repetition of the aforementioned steps. The prepared multilayer AgNR substrates were characterized by cold field-emission scanning electron microscope (JSM-6700F) at 5 kV.

### Removal and Characterization of the Surface Ligand on the AgNR Substrate

After the substrates were dried, they were immersed in an acetic acid solution for 30 s and dried at room temperature.^26^ The morphology of the surface ligand-free multilayer AgNR substrates was characterized by cold field-emission scanning electron microscope (JSM-6700F).

### Comparison and Analysis of Bacterial Identification Ability of the AgNR Substrate and Surface Ligand-Free AgNR Substrate

Two bacteria, the Gram-negative bacterium *E. coli* O157 and the Gram-positive bacterium *B. thuringiensis* (spore), with different surface compositions and structures were used to evaluate the bacterial identification ability of the multilayer AgNR substrate and the surface ligand-free multilayer AgNR substrate. After heat inactivation, *E. coli* O157 and *B. thuringiensis* (spore) were diluted to 10^7^ CFU/mL and dispersed in pure water, respectively. 10 μL of each bacterial suspension was added to both the untreated AgNR substrate (with 1 layer to 6 layers) and the acetic acid-treated AgNR substrate (with 1 layer to 6 layers), respectively, and dried at room temperature. SERS analysis of individual bacteria was performed using a HORIBA micro-convex laser Raman spectrometer (LabRAM HR Evolution) with an excitation wavelength of 633 nm, 0.5 mW laser intensity, and 5 s accumulation time. The Raman signals were collected from three different cells on the substrate, and three positions without cells were collected as blank control.

### Simulations of AgNRs

A commercial finite-element method simulation software (COMSOL Multiphysics) was applied to model the Ag nanorod dimer. A spherical domain was created around the dimer, and perfectly matched layers were employed to simulate an open boundary. To reduce the computational resources, a twofold symmetry was used. The length and the radius of the nanorods were 360 and 30 nm, respectively, which were based on the scanning electron microscopy (SEM) images of the Ag nanorods. Also, the nanogap between the Au nanorods was 3 nm. The permittivity values of the Ag nanoparticle were taken from Johnson and Christy,^27^ while the surrounding medium was set to be air, with a refractive index of *n* = 1. The incident laser wavelength was 633 nm and propagated normally to the long axis of the nanorods, while the polarization of the electric field was parallel to the nanogap.

### Bacterial Culture

The source and culture medium of these bacteria were listed in Table S1. All the bacteria were cultured and prepared for SERS measurements with the same procedures as follows: first, the strains stored in the refrigerator at −80 °C were inoculated into 3 mL liquid culture medium and activated at a suitable temperature to prepare as seed liquid for further use. Then, 20 μL of seed liquid was added to 5 mL liquid medium and cultivated at 37 °C for 12 h. Next, the culture medium was removed by centrifugation at 7000 rpm for 5 min with 1 mL of the cultured bacterial liquid, and then, 1 mL of 8% normal saline was added to resuspend and centrifuge at 7000 rpm for 5 min to remove the supernatant. This step was repeated three times. Afterward, the bacteria were resuspended in 200 μL of 8% normal saline and inactivated at 65 °C for 30 min. Before SERS measurements, the inactivated bacteria were washed three times with ultrapure water.

### Bacterial Identification Generality Determination

The generality of the developed SERS sensor was evaluated by using the optimized surface ligand-free single-layer AgNR substrate to analyze 22 kinds of bacteria (12 kinds of Gram-negative bacteria, 9 kinds of Gram-positive bacteria, and 1 kind of acid-fast staining bacteria). All bacteria were heat-inactivated, diluted to 10^7^ CFU/mL, and dispersed in pure water. 10 μL of each was pipetted onto the substrate and dried at room temperature. SERS analysis of individual bacteria was performed using a HORIBA micro-convex laser Raman spectrometer (LabRAM HR Evolution) with an excitation wavelength of 633 nm, 0.5 mW laser intensity, and 5 s accumulation time. The Raman signals of three different cell individuals were collected for each bacterium, and three positions without cells were collected as blank controls.

### Statistical Data Analysis

First, the original Raman spectra were normalized using probabilistic quotient normalization (PQN). Following normalization, *t*-test analysis using MetaboAnalyst 4.0 (https://www.metaboanalyst.ca) was carried out to identify 5 features with the highest significance discriminating the spectra of a given strain from the spectra of the collection of the remaining 21 bacterial strains. The significance level in all analysis was considered to be *P* ≤ 0.05. Subsequently, a region of ±5 cm^–1^ (Figures S8–S29A) around these features (frequencies) was integrated. Differences of these features (integrals and peak ratios) between a given strain and remaining 21 bacterial strains were assessed by a two-tailed Student’s *t*-test for pairwise comparison (Figures S8–S29B). Integrals and peak ratios of features were used to perform a univariate receiver operating characteristic (ROC) curves analysis by calculating their 95% confidence intervals in MetaboAnalyst 4.0 (Figures S8–S29C). The optimal threshold (best cut-off value) was determined according to the sensitivity and specificity. Significance was set at *P* < 0.05. Statistical analysis and graphs were generated using GraphPad Prism 5.01. software (GraphPad Software, La Jolla, CA, USA)

## Results and Discussion

### Characterization of AgNRs

**Figure 1:**
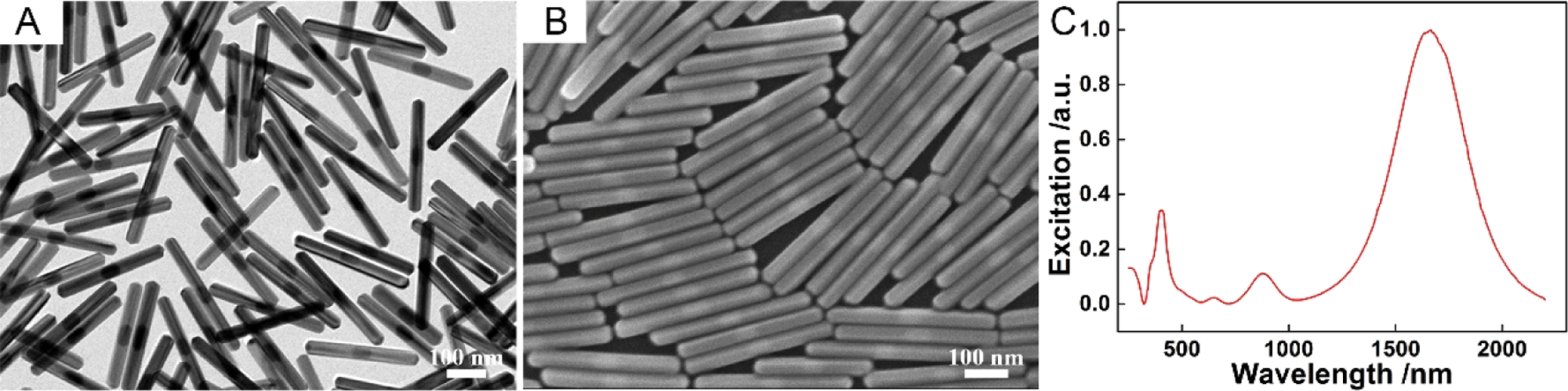
Characterizations of AgNRs of (A) transmission electron microscopy, (B) SEM, and (C) UV–vis–IR spectra.

AgNRs were synthesized with the method proposed by our group before,^24,25^ with a length of 357 ± 14 nm and a width of 31 ± 1 nm, as can be seen in Figures 1A,B and S1. The UV–vis–IR spectrum of AgNRs shown in Figure 1C shows the longitudinal dipolar plasmon resonance at 1665 nm and transverse dipolar plasmon resonance at 401 nm. Two well-resolved peaks at 648 and 880 nm between the transverse and longitudinal dipolar plasmon modes appear, indicating the homogeneity of AgNRs.^28,29^ The good monodispersity and uniformity of these AgNRs make them ideal candidates as building blocks.

### Optimization of the Bacterial-Oriented AgNR SERS Substrate

Here, Gram-negative *E. coli* O157 and Gram-positive *B. thuringiensis* (spore) are chosen as model systems. These strains have significant differences concerning their surface structures and chemical compositions and thus represent two extreme situations for the bacteria strains.^30^ It is known that the number of layers (NL) of the substrate alters the SERS performance.^29^ To optimize the SERS sensor for pathogenic bacteria differentiation, the influence of NL was studied. Self-assembly of AgNRs on the air–liquid interface with horizontal orientations was carried out to form a uniform AgNRs layer.^28,29^ By simply repeating this assembly process, one can easily obtain the SERS substrate with varying AgNR NL.

**Figure 2:**
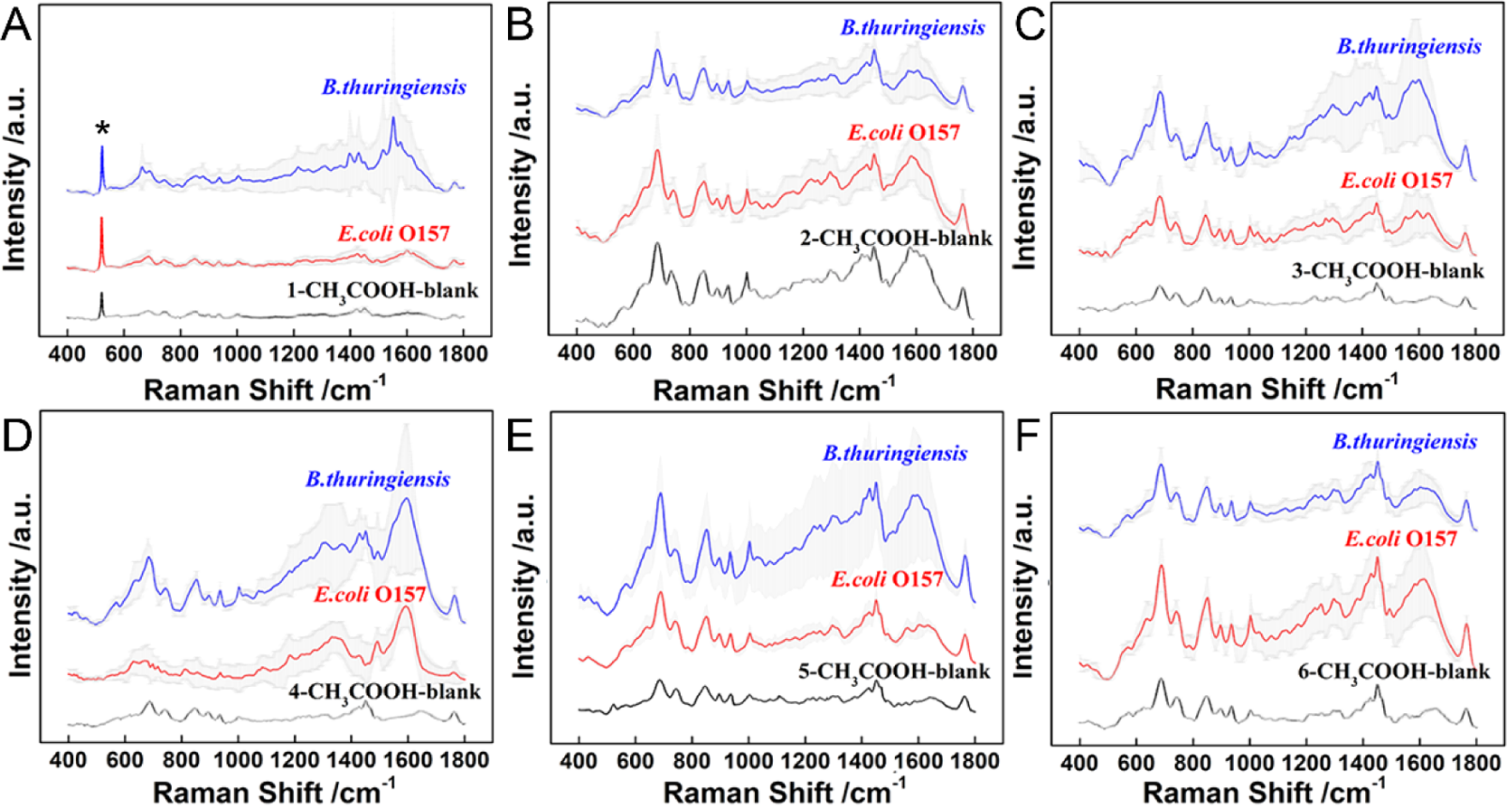
Mean average SERS spectra of blank and bacteria with different layers after (A–F) acetic acid treatment. (A) NL = 1, (B) NL = 2, (C) NL = 3, (D) NL = 4, (E) NL = 5, and (F) NL = 6. Gray color shows the error bar of each spectra. The error bar is based on three spectra, namely, three individual bacteria were tested and each bacterium was measured to generate one SERS spectra. The peak marked with * indicates the Si peak.

Moreover, to remove the surfactant agent residue (PVP), we adopted an acetic acid treatment method^26^ for the SERS substrate. Regarding the removal mechanism, first, acetic acid helps to remove the surface metal oxide, which simultaneously leads to the detachment of surface PVP.^31^ In addition, PVP could easily dissolve in acetic acid.^32^ Both factors contribute to remove PVP. It can be seen in Figure S2 that, without the acetic acid treatment, for all NLs from one to six, the spectra obtained from the SERS substrate do not show much difference for two model bacteria. It is much likely due to the existence of the PVP layer on the AgNR surface, which hinders the direct adsorption of bacteria onto AgNRs and thus compromises the detection sensitivity. Intriguingly, after the removal of PVP with the treatment of acetic acid, the SERS substrate with the AgNR monolayer (NL = 1) provides the largest difference of Raman features ranging from 1200 to 1800 cm^–1^, as presented in Figure 2A. However, with the NL increasing, these differences vanish as observed in Figure 2B–F. We deduce that for a thick AgNR layer, it is more difficult to remove the PVP residue inside the 3D structure, especially the hot spot, and therefore results in a stronger background, which deteriorates the signal-to noise ratio of the Raman signal of target bacteria and decreases the sensitivity of the SERS substrate. More importantly, before the treatment of acetic acid, the statistical analysis (Figure S3A) showed that there was no significant difference (*p* > 0.001). Nevertheless, after the treatment of acetic acid, the significant difference (*p* < 0.001) was clearly observed (Figure S3B), indicating the necessity of the treatment with acetic acid. The results demonstrated the effective treatment of acetic acid. Additionally, the SERS substrate with the AgNR monolayer shows good homogeneity with a 6% relative standard deviation value (Figure S7). To examine the EM field enhancement effect of this SERS substrate under illumination, theoretical calculations were performed, as presented in Figure S6C.^33,34^ It can be clearly seen that the hot spot is located between two Ag NRs, and the overall EM enhancement |*E*/*E*_0_|^4^ is as high as 4.45 × 10^5^. Taken all, the SERS substrate with the AgNR monolayer provides the highest sensitivity and feasibility for bacteria identification.

### Generality Evaluation of the AgNR SERS Substrate Using the Statistical Method

To determine the generality of the optimized SERS substrate for a wide range bacteria identification with different staining categories, morphologies, and species classifications, 22 types of bacteria were further explored, including 4 types of biological warfare agent pathogens, 1 type of strong infectious disease-related pathogen, and 7 types of food safety-related pathogens. Regarding staining classification, there are 12 types of Gram-negative, 9 types of Gram-positive, and 1 type of acid-fast staining bacteria. In terms of morphological classification, they can be divided into 2 types of coccus, 18 kinds of bacillus, 1 kind of vibrio, and 1 kind of mycobacterium. Considering species classification of five types of *Bacillus* spp. and seven types of *Salmonella* spp. are included. Again, this study covers a wide range of the most common pathogenic bacteria in the daily life.

**Figure 3:**
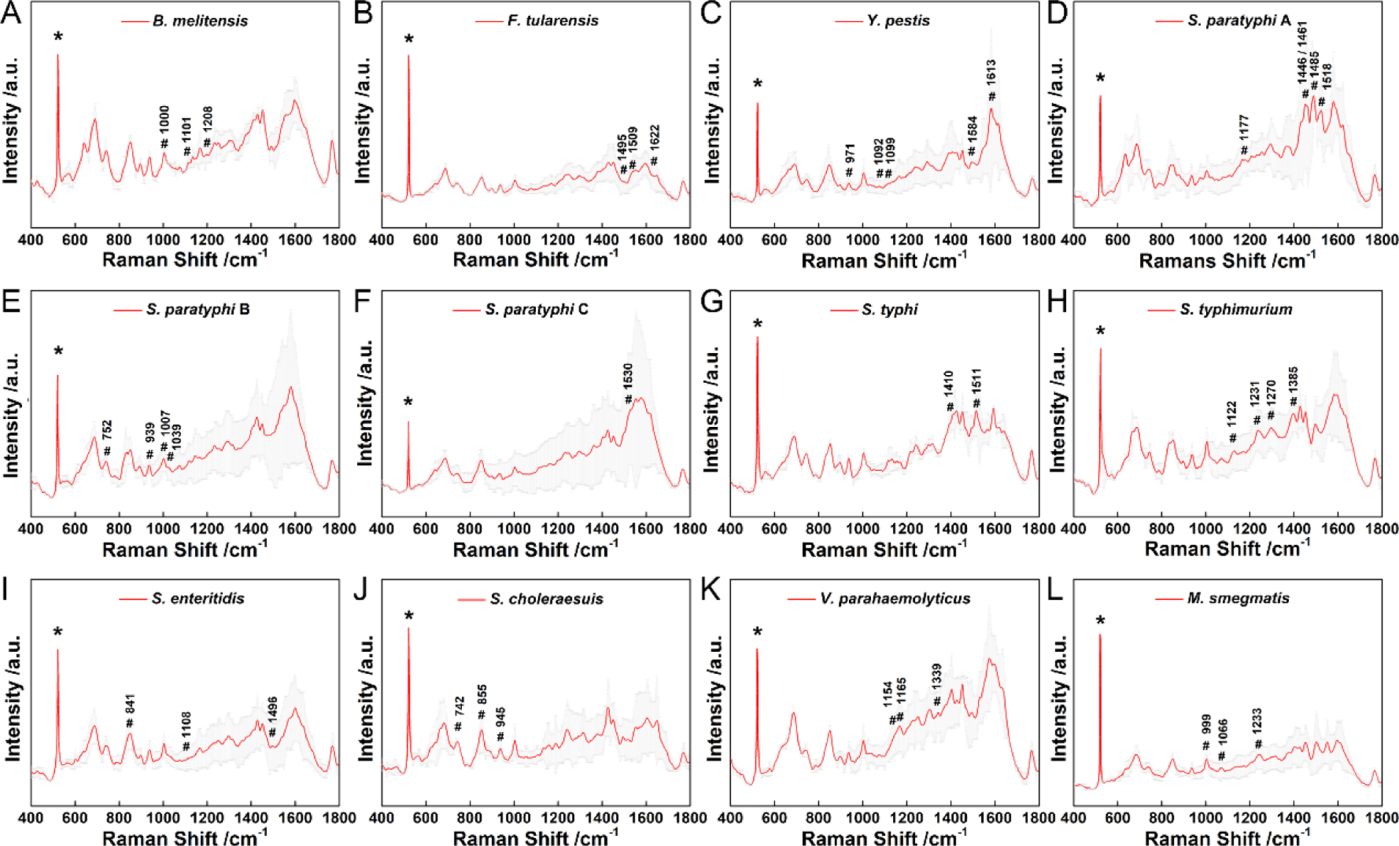
SERS spectra of Gram-negative bacteria of (A) *B. melitensis*, (B) *F. tularensis*, (C) *Y. pestis*, (D) *S. paratyphi* A, (E) *S. paratyphi* B, (F) *S. paratyphi* C, (G) *S. typhi*, (H) *S. typhimurium*, (I) *S. enteritidis*, (J) *S. choleraesuis*, (K) *V. parahaemolyticus* and acid-fast staining bacteria (L) *M. smegmatis*. Gray color shows the error bar of each spectrum. The peak marked with * indicates the Si peak. The peaks marked with # indicate the Raman features obtained from *t*-test analysis, which can be assigned to the vibrational modes of biomolecules in Table S3.

**Figure 4:**
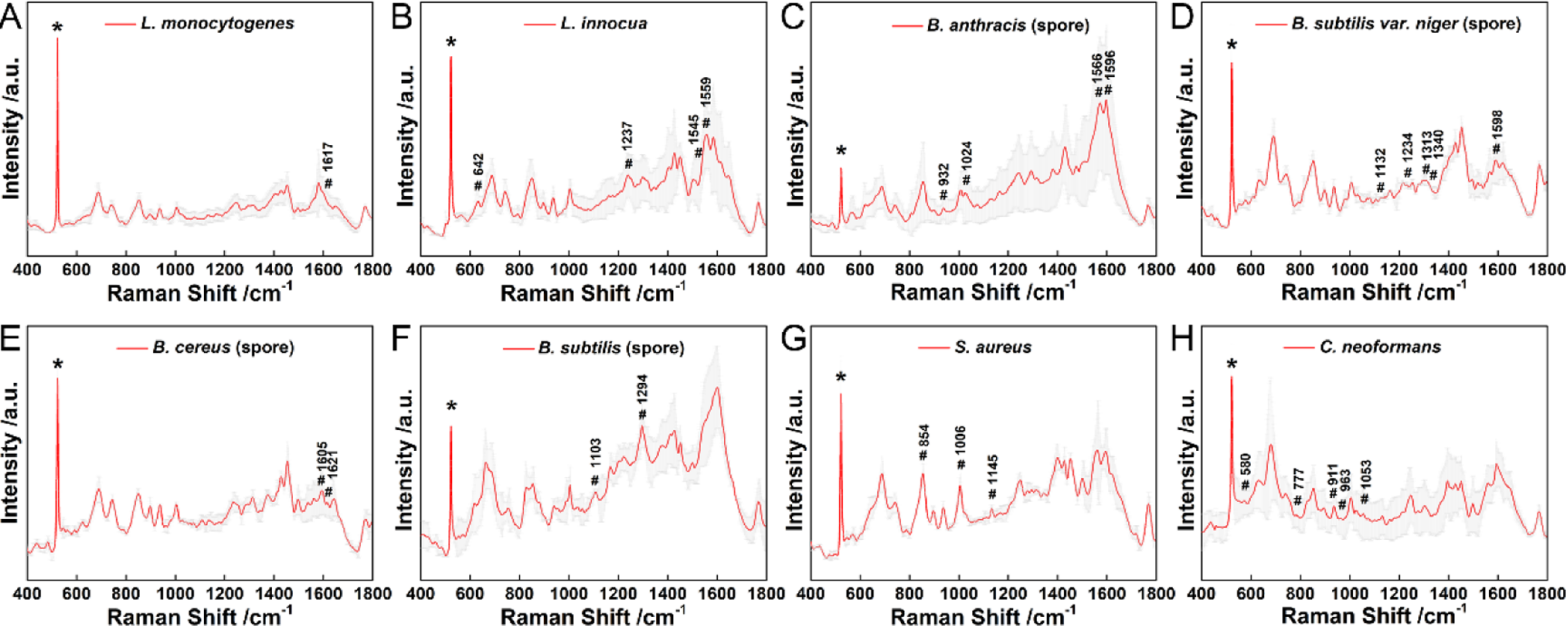
SERS spectra of Gram-positive bacteria of (A) *L. monocytogenes*, (B) *L. innocua*, (C) *B. anthracis* (spore), (D) *B. subtilis*var. *niger* (spore), (E) *B. cereus* (spore), (F) *B. subtilis* (spore), (G) *S. aureus*, and (H) *C. neoformans*. Gray color shows the error bar of each spectrum. The peak marked with * indicates the Si peak. The peaks marked with # indicate the Raman features obtained from *t*-test analysis, which can be assigned to the vibrational modes of biomolecules in Table S3.

**Figure 5:**
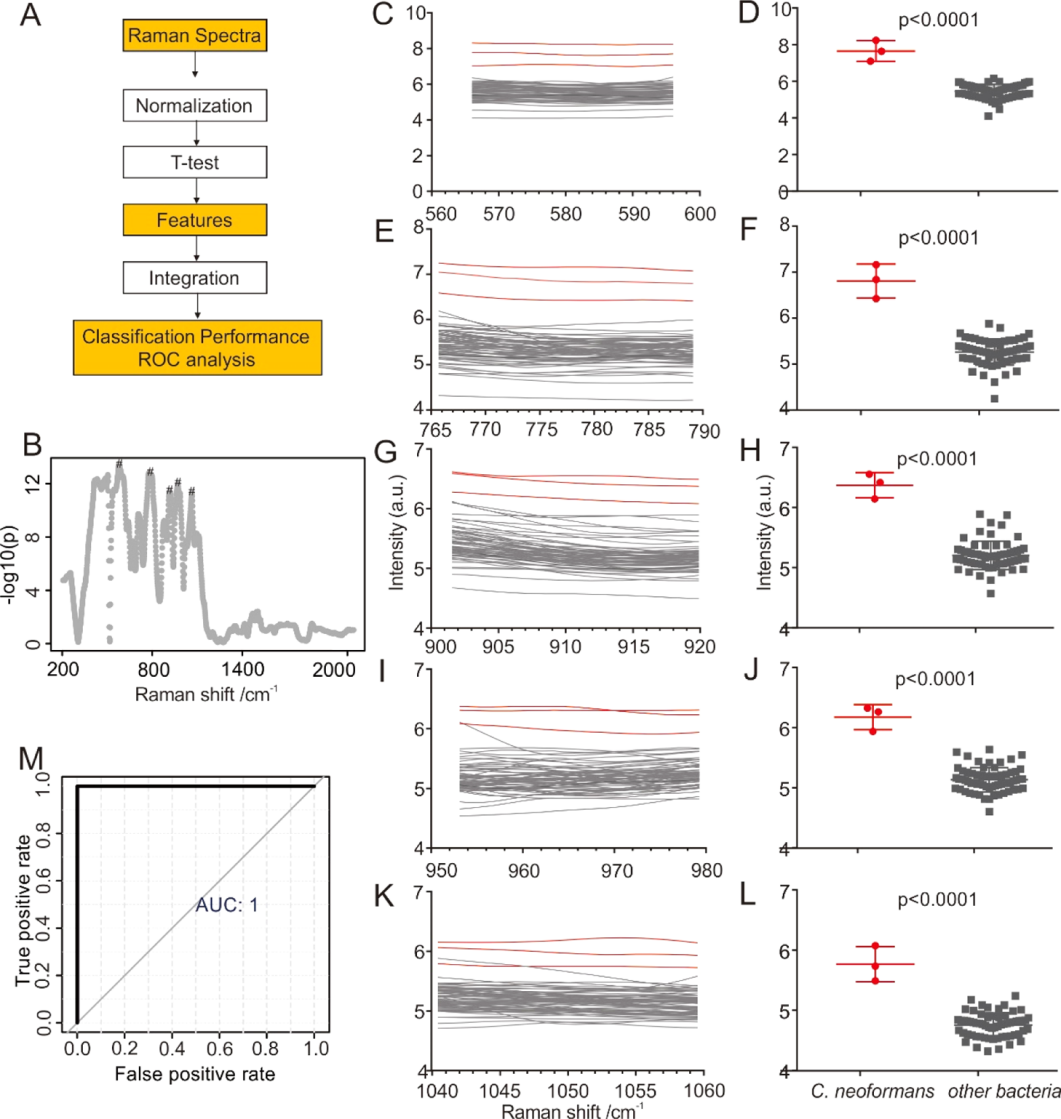
(A) Schematic of the SERS data analysis pipeline. The details are as follows: The SERS spectra used as functions of the Raman frequency were normalized by MetaboAnalyst 4.0, using PQN. A *t*-test was used to unveil 5 features with the highest significance discriminating the spectra of a given strain from that of the collection of the remaining 21 bacterial strains. The corresponding SERS spectra of these five regions (features) ±5 cm^–1^ around these frequencies were integrated, and then, the integrals or peak ratios were used as the criteria to determine the diagnostic capabilities (ROC analysis). (B) Differences of *C. neoformans* SERS spectra as a function of the Raman frequency compared to the pool of the 21 bacteria *t*-test. The analysis was performed using MetaboAnalyst 4.0. This plot has been used to identify the five regions with the best discriminatory performance (labeled with an asterisk). The corresponding SERS spectra (C,E,G,I,K) and normalized intensities (D,F,H,J,L) of these five regions are shown on the right. The spectra of *C. neoformans* were marked with a red line, and other bacteria were marked with a gray line. (M) Comparison of integrals used in the ROC analysis.

The reference spectra map corresponding each bacterium was generated from the pattern of vibrational bands. Taken staining classification into consideration, all the SERS spectra of different bacteria are shown in Figures 2A–4 (12 Gram-negative bacteria, 1 acid-fast staining bacterium, and 9 Gram-positive bacteria). The obtained spectra differ from each other, and statistical methods were utilized to unravel the intrinsic difference. In this context, the analysis was performed over the entire spectral region between 200 and 2000 cm^–1^. The detailed data processing procedures are shown in Figure 5A, based on SERS fingerprint signals and statistical methods. Take *C. neoformans,* for example, a bacterium that can cause cryptococcal meningoencephalitis,^35,36^ the distinct Raman features are retrieved as labeled with asterisk in Figure 5B. The corresponding frequencies are listed in Figure 5C–L and Table S2. We first normalized the Raman spectra using PQN, an approach which is widely used in metabolomics. PQN has been proven robust and accurate, in particular, in cases of complex spectral features, such as the biological Raman spectra.^37^ The SERS spectra used as functions of the Raman frequency were normalized by MetaboAnalyst 4.0. After normalization, a *t*-test analysis was used to unveil 5 features with the highest significance discriminating the spectra of a given strain from that of the collection of the remaining 21 bacterial strains (Figure 5B). The corresponding SERS spectra of these five regions (features) (Table S2) ±5 cm^–1^ around these frequencies were integrated (Figure 5C,E,G,I,K), and then, the integrals or peak ratios were used as the criteria to examine whether the spectral features of the given strain are significantly different from the remaining 21 bacterial strains (Figure 5D,F,H,J,L). In the end, these values are employed in ROC analysis to determine the diagnostic capabilities (Figure 5M).

Subsequently, a region of ±5 cm^–1^ around these frequencies was integrated, and then, the integrals or peak ratios were used as the criteria to determine the diagnostic capabilities. The integrals of the corresponding five regions of all the bacteria are presented in Figure 5D,F,H,J,L. It can be seen that, indicated by all the Raman features, *C. neoformans* can be clearly distinguished from the rest of bacteria, with all *p* values less than 0.0001. In the end, the ROC analysis was applied to evaluate the classification performance. As shown in Figure 5M, areas under the curve (AUC) of the ROC curve is determined to be 1, validating the outstanding performance of this classification method. All the five regions with the best distinguished features shown in Figure 5B correspond to the vibrations of the biological functional group. Specifically, the bands at 580, 777, 911, 963, and 1053 cm^–1^ can be assigned to C–O–C glycosidic ring deformation, adenine (as well as FAD and NAG), C–C stretching modes in proteins, C–N stretching modes, and C–C ring breathing.^38−40^ Therefore, the SERS classification is based on the intrinsic difference of biological compositions of bacteria.

Thrillingly, as exhibited in Table S2, Figures 5, and S8–S29, 20 out of 22 bacterial strains could be discriminated, as indicated by the AUC value, which is close to 1. The spectral features with the best performance are shown in Table S2, and the major vibrational modes of bacteria, like nucleic acids, carbohydrates, and lipids are listed in Table S3. Unfortunately, two strains, *Salmonella enteritidis* and *L. innocua*, could not be discriminated, whose AUC value is less than 0.804 and 0.738, respectively. It is considered that the biomolecules on these two strains share the same fingerprint information with the rest of other bacteria without abundant distinct biological components, but additional investigations are needed.

Moreover, this method can also be applied in the identification of the mixture of many species of bacteria. In Figure S30A, the mixture of bacteria comprising six pathogens (*M. smegmatis*, *S. aureus*, *E. coli* O157, *S. paratyphi* A, *V. Parahaemolyticus*, and *L. monocytogenes*) was spread on the SERS substrate. According to the morphological classification, all the six pathogens can be divided into four categories: *Mycobacterium* (*M. smegmatis*), *Coccus* (*S. aureus*), bacterium (*E. coli* O157, *S. paratyphi* A, and *L. monocytogenes*), and *Vibrio* (*V. Parahaemolyticus*). It should be noted that based on the morphology in the optical image (Figure S30A), it is hard to tell the difference between bacterium and *Vibrio*. Therefore, as shown in Figure S30A, both bacterium and *Vibrio* are indicated with a blue circle, while Mycobacterium is marked in red and Coccus was marked in yellow. Taking *M. smegmatis* (indicated with a red circle) as an example, the same method used in the paper was also performed to distinguish *M. smegmatis* from the mixture of other five bacteria. The *t*-test was used to unveil five features with the highest significance discriminating the spectra of *M. smegmatis* from that of the collection of the remaining five bacterial strains. Then, the corresponding SERS spectra of these five regions (features) ±5 cm^–1^ around these frequencies were integrated, and the integrals were used as the criteria to determine the difference of these bacteria (Figure S30B) and the diagnostic capabilities (Figure S30C). As shown in Figure S30B, the value of *p* is smaller than 0.0001, indicating the significant difference between *M. smegmatis* (red dot, Figure S30B) and other bacteria (gray dot, Figure S30B). In addition, the AUC value of the ROC curve with a 95% confidence interval (Figure S30C) is 0.98, demonstrating the reliability of this method. These results prove that this strategy provides a reliable methodology for bacteria identification.

## Conclusions

In summary, we rationally design a novel SERS substrate for the wide-range, label-free, rapid, and specific detection of most common pathogenic bacteria. The optimized AgNR substrate provided abundant fingerprint information, which was then successfully used for the differentiation of 22 kinds of pathogenic bacteria, including the biological warfare agent and strong infectious disease-related and food safety-related pathogens. The multivariate procedures were applied to achieve accurate diagnosis based on the SERS spectra of bacteria. It showed that 20 out of 22 bacterial strains was discernible with high specificity and sensitivity by statistical analysis. The high accuracy of discrimination of pathogenic bacteria by SERS makes it possible to detect the pathogens in a short time (no more than 30 min). These results help to expand the generality of the SERS sensor for bacteria identification, which promises the future point-of-care testing-based applications.

## Supporting Information Available

Statistical diagram of AgNRs, SEM and comparison of AgNR substrates, comparison of statistical analysis before and after acetic acid, image of simulation of AgNRs, SERS intensity of the monolayer AgNR substrate, SERS spectra of bacteria, statistical analysis of other 21 bacteria, statistical analysis of bacteria mixture, source of bacteria, table of AUC values obtained from ROC analysis for all bacteria, and table of major spectral band assignments for bacteria (PDF)
