# Supplementary material for: Wide-Range, Rapid, and Specific Identification of Pathogenic Bacteria by Surface-Enhanced Raman Spectroscopy
Source: ACS Sens. 2021 Jul 20;6(8):2911–9. doi: 10.1021/acssensors.1c00641 (PMC8406416; doi:10.1021/acssensors.1c00641)
Supplement: Supplementary file 1 — se1c00641_si_001.pdf [file se1c00641_si_001.pdf]

Supporting Information

Wide-Range, Rapid, and Specific Identification of Pathogenic Bacteria by Surface-Enhanced Raman Spectroscopy

Siying Liu,[‡,1,2,3] Qiushi Hu,[‡,4] Chao Li,[‡,5] Fangrong Zhang,[6,7] Hongjing Gu,[8] Xinrui Wang,[9] Shuang Li,[4] Lei Xue,[4] Tobias Madl,[1,2,6,7,*] Yun Zhang,[1,2,3,*] Lei Zhou,[4,*]

[1] CAS Key Laboratory of Design and Assembly of Functional Nanostructures, and Fujian Provincial Key Laboratory of Nanomaterials, Fujian Institute of Research on the Structure of Matter, Chinese Academy of Sciences, Fuzhou 350002, China

[2] Department of Translational Medicine, Xiamen Institute of Rare Earth Materials, Chinese Academy of Sciences, Xiamen 361021, China

[3] University of Chinese Academy of Sciences, Beijing 100049, China

[4] State Key Laboratory of Biochemical Engineering, PLA Key Laboratory of Biopharmaceutical Production & Formulation Engineering, Institute of Process Engineering, Chinese Academy of Sciences, Beijing 100190, China

[5] Institute of Medical Equipment, Academy of Military Sciences, Tianjin 300161, China

[6] Gottfried Schatz Research Center for Cell Signaling, Metabolism and Aging, Institute of Molecular Biology & Biochemistry, Medical University of Graz, Neue Stiftingtalstrasse 6, 8010 Graz, Austria

[7] BioTechMed-Graz, Graz, Austria

[8] State Key Laboratory of Pathogen and Biosecurity, Beijing Institute of Microbiology and Epidemiology, Beijing 100071, China

[9] Anti-plague Institute Hebei Province, Zhangjiakou 075000, China

[‡] Equal contribution

* Corresponding authors, Email: tobias.madl@medunigraz.at, zhangy@fjirsm.ac.cn, zhoulei17@ipe.ac.cn

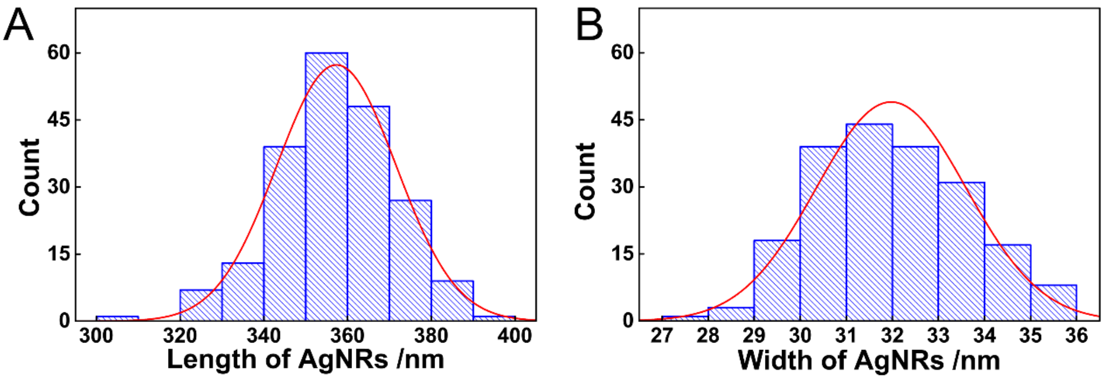

Figure S1. Statistical diagrams of the length (A) and width (B) of AgNRs.

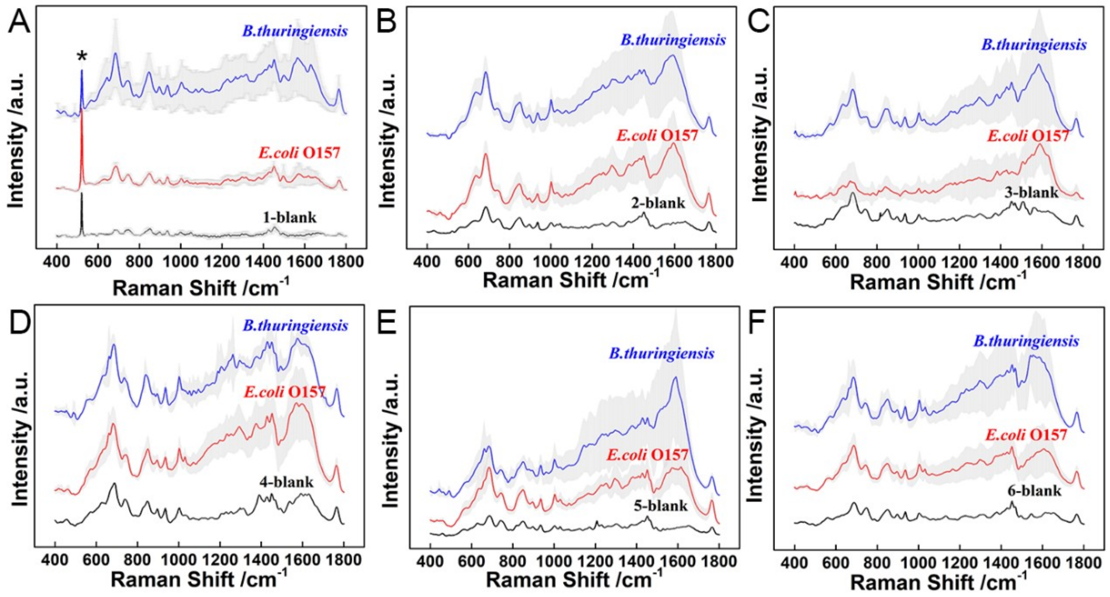

Figure S2. Mean average SERS spectra of blank and bacteria with different layers before (A-F) acetic acid treatment. (A) NL = 1, (B) NL = 2, (C) NL = 3, (D) NL = 4, (E) NL = 5, (F), NL = 6. Gray color shows the error bar of each spectra. The peak marked with * indicates Si peak.

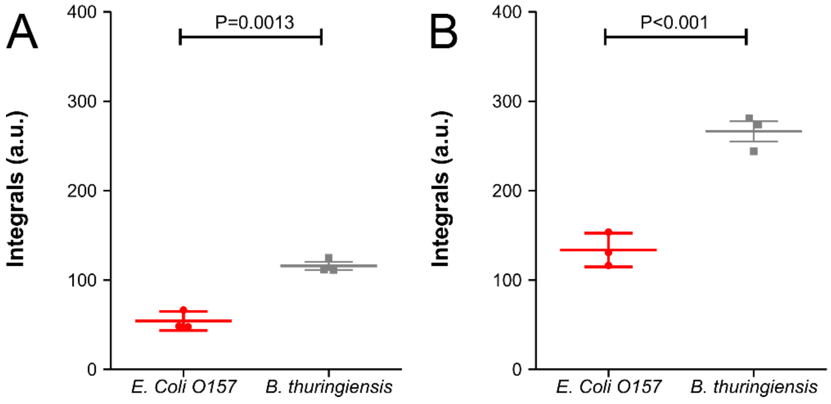

Figure S3. The integral of Raman shift: 1769 for (before acetic acid) and 526 for (after acetic acid) in the *E. Coli* O157 (red) and *B. thuringiensis* (grey), respectively.

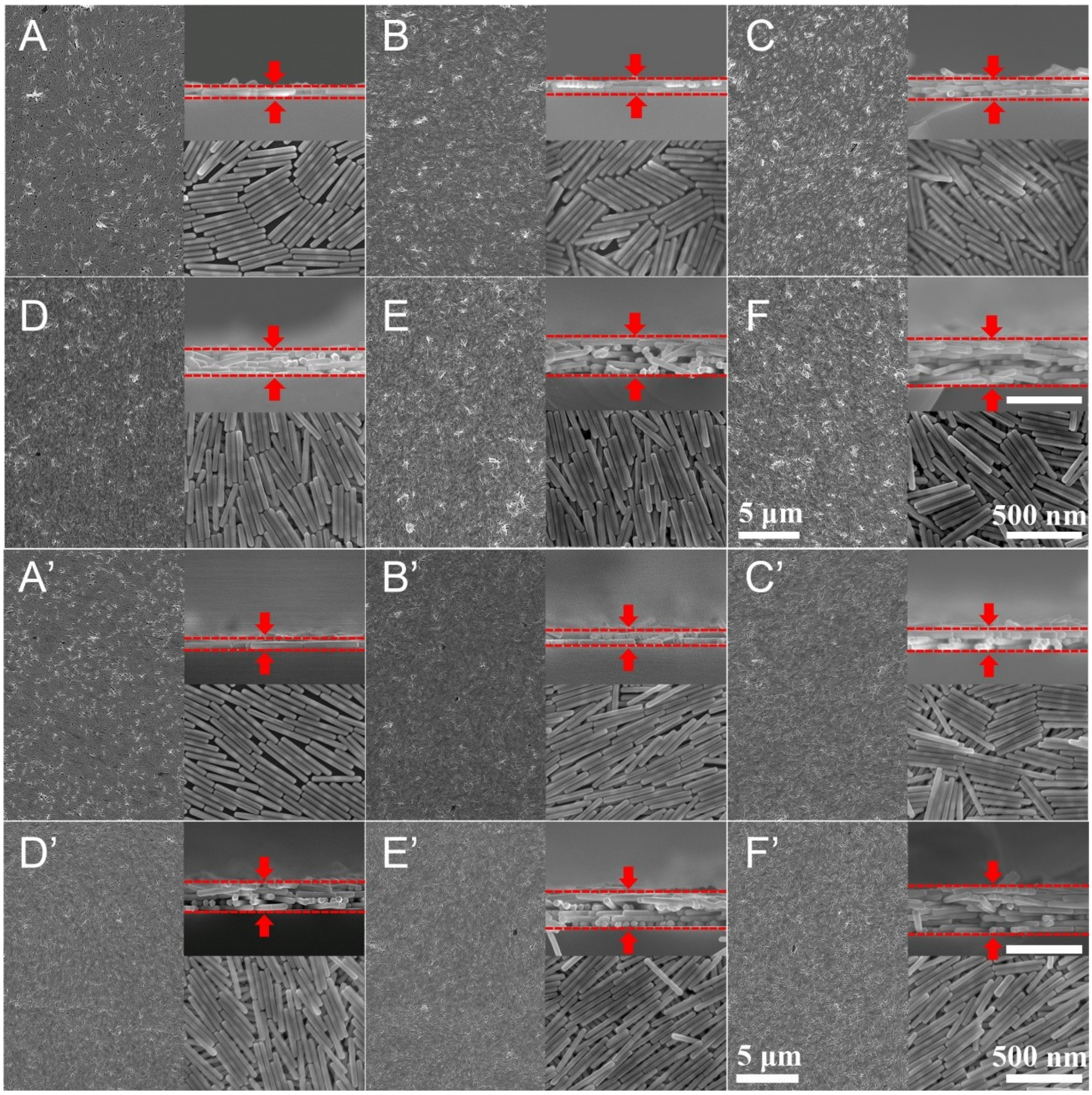

Figure S4. SEM of SERS substrates with different number of layers (NL) of untreated (A-F) and acetic acid treated (A'-F') substrate and the diagram of the relationship between the NL and thickness of the substrates. (A), (A') NL = 1, (B), (B') NL = 2, (C), (C') NL = 3, (D), (D') NL = 4, (E), (E') NL = 5, (F), (F') NL = 6.

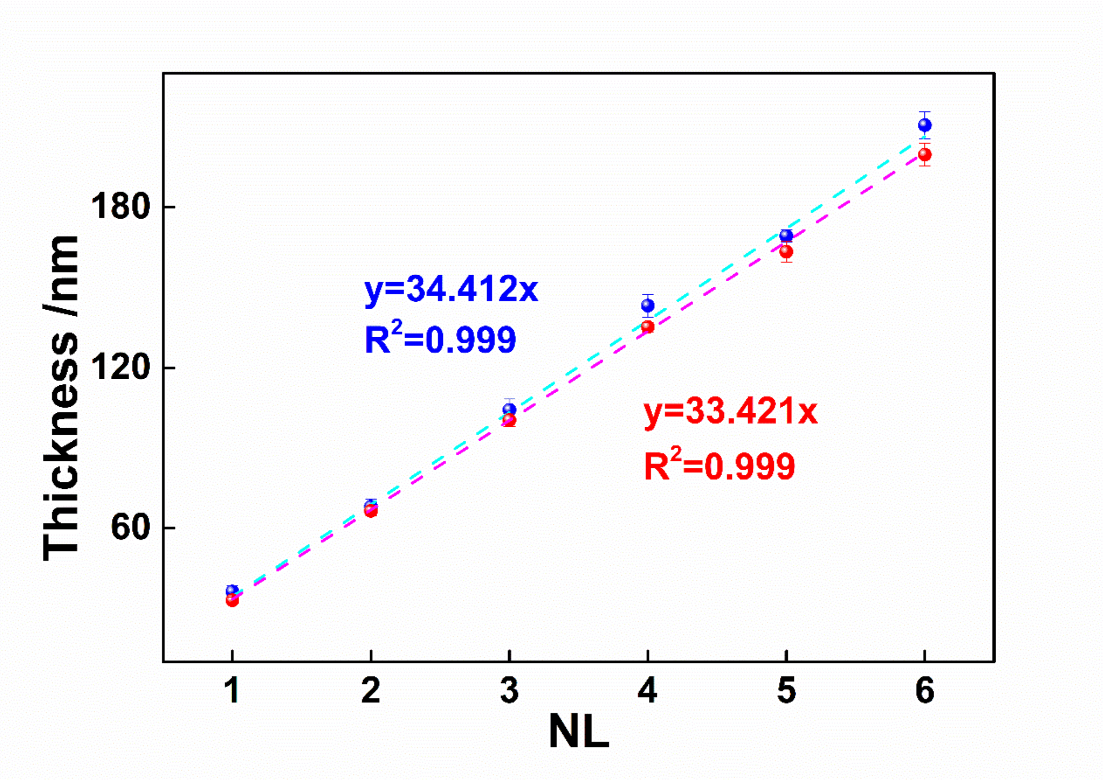

Figure S5. Relationship of NL and thickness of the substrates (the untreated marked in blue, the acetic treated marked in red). It can be seen that after the treatment of acetic acid, the thickness of SERS substrate is slightly thinner than the original one, indicating the partial removal of PVP on AgNRs.

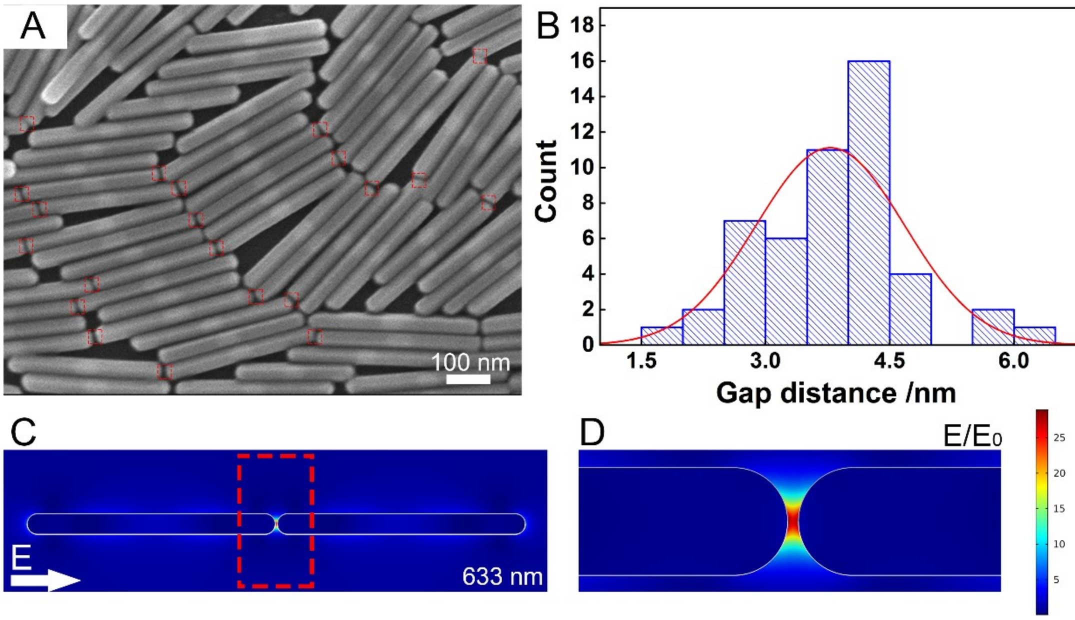

Figure S6. (A) SEM image of AgNRs. The distance calculated between the two ends of AgNRs are marked in red, dashed box. (B) Statistical diagram of the gap distance between two ends of AgNRs calculated from (A). (C) Theoretical simulation of the electromagnetic field enhancement between two AgNRs. The length of AgNRs was 360 nm, and the gap between two

AgNRs was 3 nm. The excitation laser wavelength was 633 nm. The intensity of the electromagnetic field is represented by color scale. (D) Zoom of the hot spot between two AgNRs of (C).

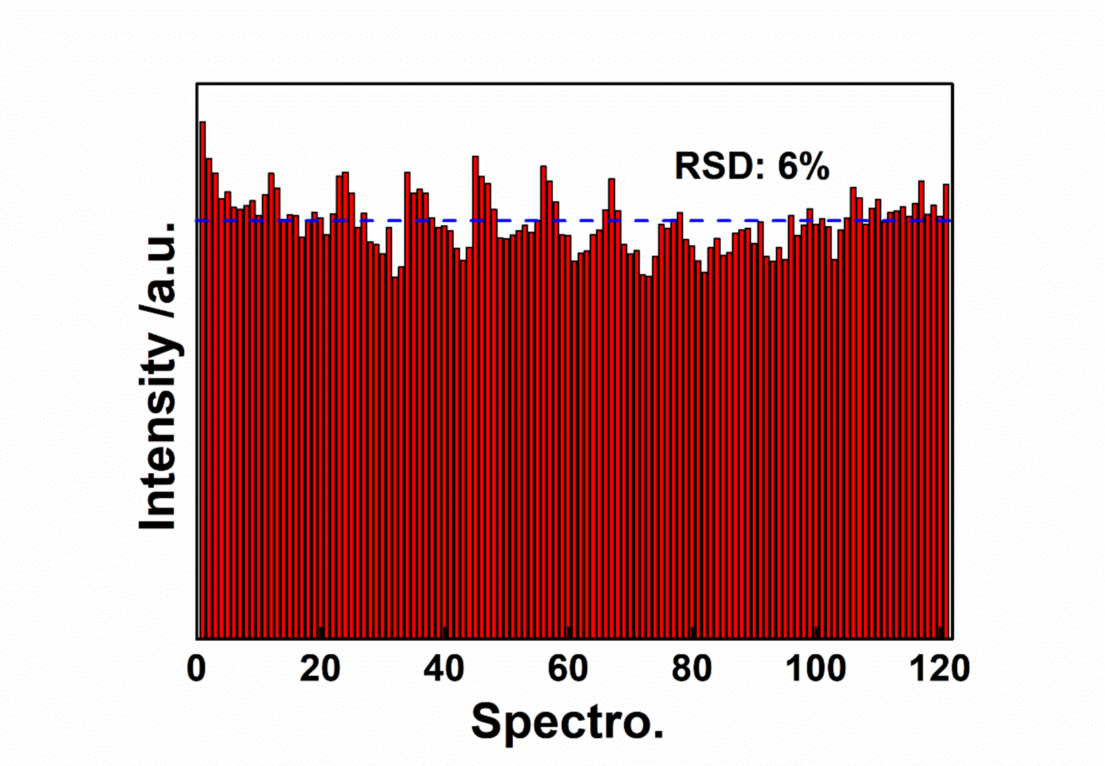

Figure S7. SERS intensity of the monolayer AgNRs substrate, showing a relative standard deviation of 6%.

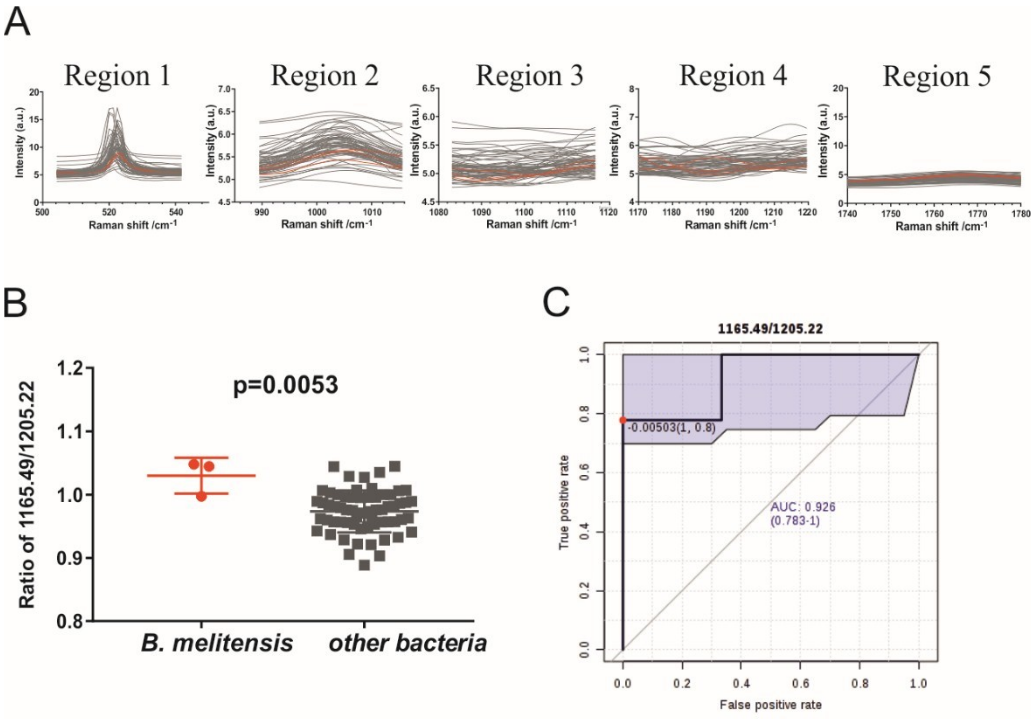

Figure S8. (A) SERS spectra of different region (selected) between the *B. melitensis* (red) and other 21 bacteria (grey). (B) Ratio of integral of Raman shift: 1165.49/1205.22 in the *B. melitensis* (red) and other 21 bacteria (grey). (C) ROC curve with a 95% confidence interval (in brackets) generated from defined feature (ratio of integral of Raman shift: 1165.49/ 1205.22).

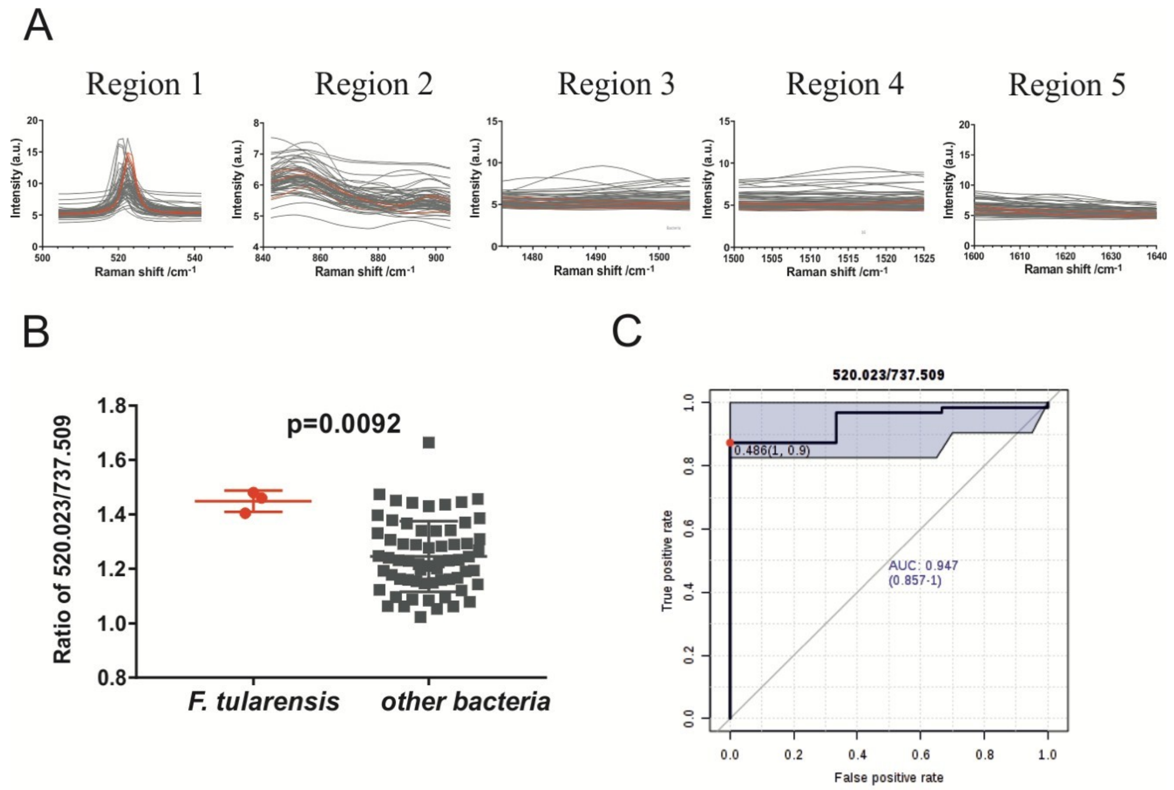

Figure S9. (A) SERS spectra of different region (selected) between the *F. tularensis* (red) and other 21 bacteria (grey). (B) Ratio of integral of Raman shift: 520.023/ 737.509 in the *F. tularensis* (red) and other 21 bacteria (grey). (C) ROC curve with a 95% confidence interval (in brackets) generated from defined feature (ratio of integral of Raman shift: 520.023/ 737.509).

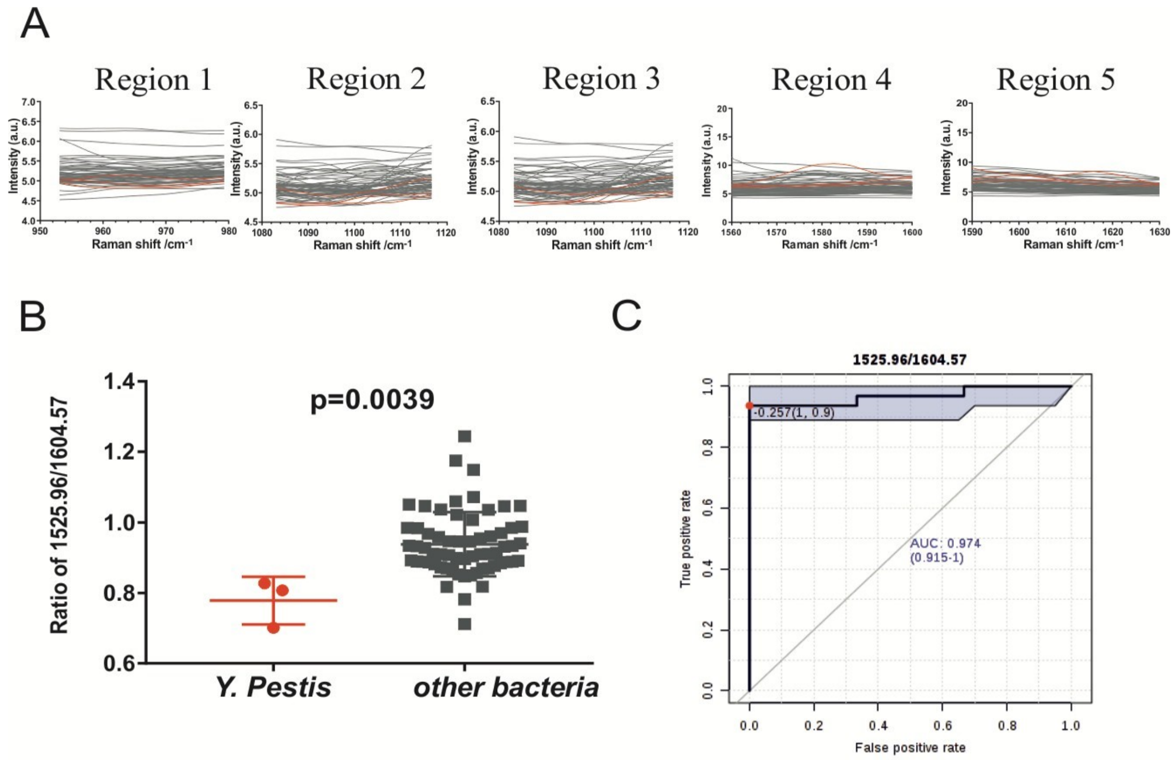

Figure S10. (A) SERS spectra of different region (selected) between the *Y. pestis* (red) and other 21 bacteria (grey). (B) Ratio of integral of Raman shift: 1525.96/ 1604.57 in the *Y. pestis* (red) and other 21 bacteria (grey). (C) ROC curve with a 95% confidence interval (in brackets) generated from defined feature (ratio of integral of Raman shift: 1525.96/ 1604.57).

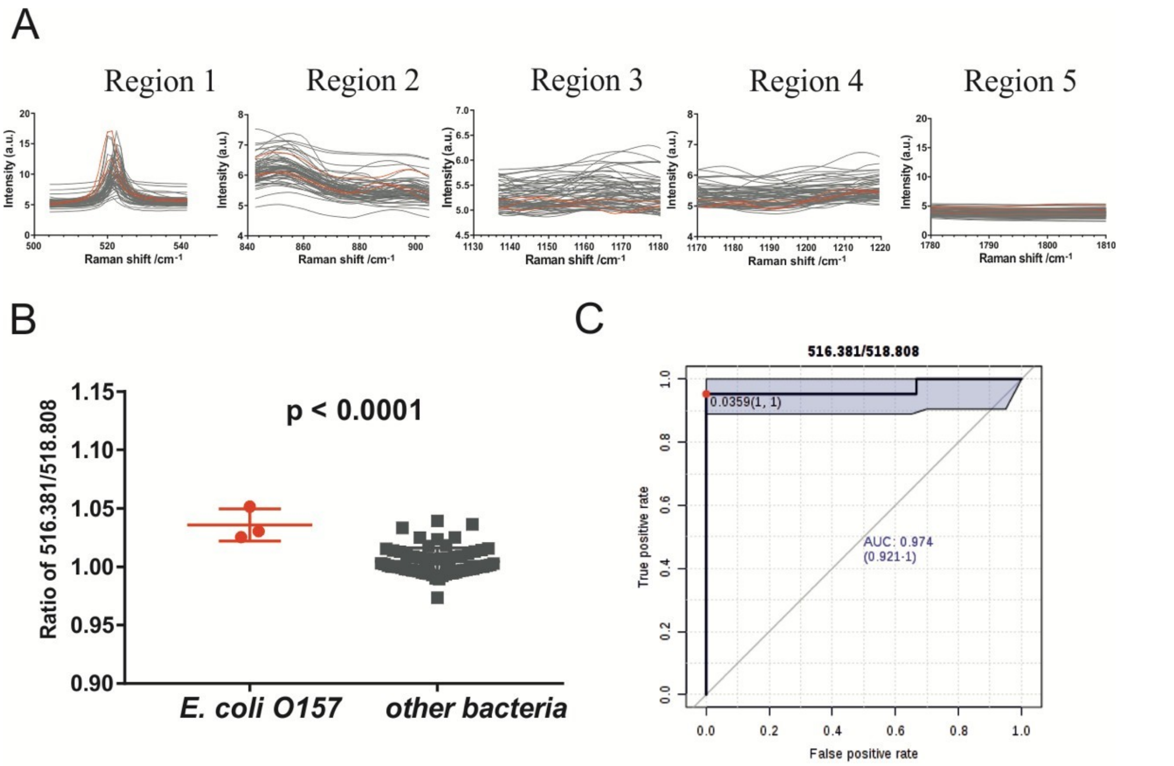

Figure S11. (A) SERS spectra of different region (selected) between the *E. coli* O157 (red) and other 21 bacteria (grey). (B) Ratio of integral of Raman shift: 516.381/ 518.808 in the *E. coli* O157 (red) and other 21 bacteria (grey). (C) ROC curve with a 95% confidence interval (in brackets) generated from defined feature (ratio of integral of Raman shift: 516.381/ 518.808).

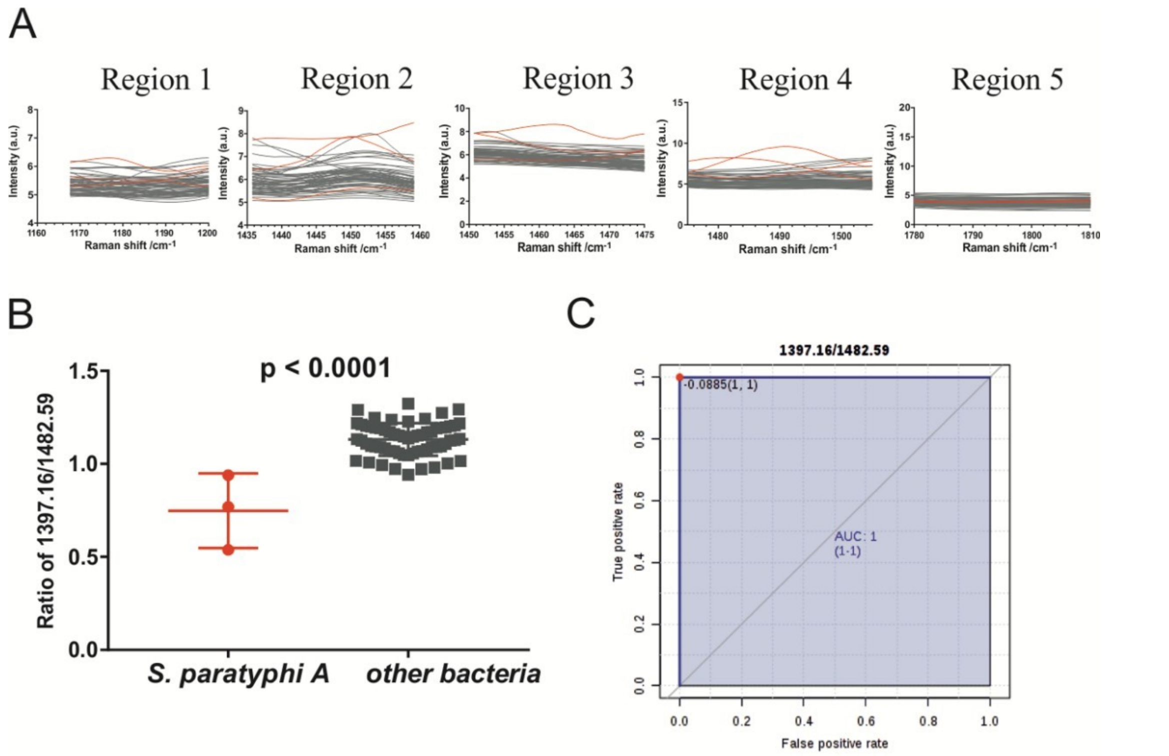

Figure S12. (A) SERS spectra of different region (selected) between the *S. paratyphi* A (red) and other 21 bacteria (grey). (B) Ratio of integral of Raman shift: 1397.16/ 1482.59 in the *S. paratyphi* A (red) and other 21 bacteria (grey). (C) ROC

curve with a 95% confidence interval (in brackets) generated from defined feature (ratio of integral of Raman shift: 1397.16/ 1482.59).

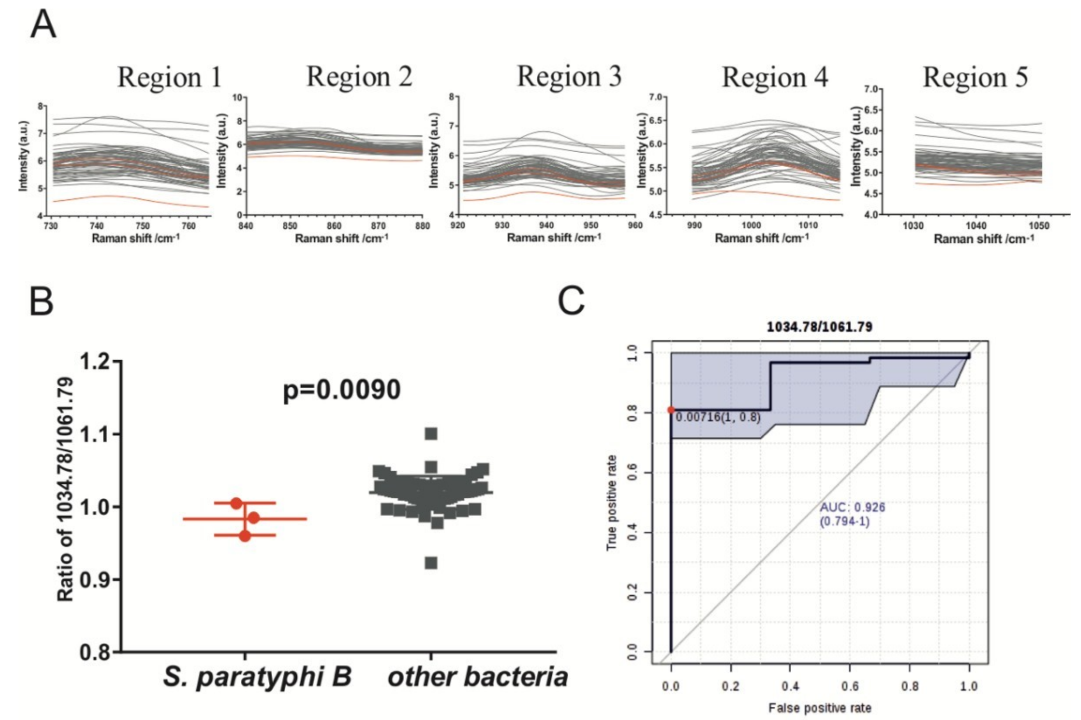

Figure S13. (A) SERS spectra of different region (selected) between the *S. paratyphi* B (red) and other 21 bacteria (grey). (B) Ratio of integral of Raman shift: 1034.78/ 1061.79 in the *S. paratyphi* B (red) and other 21 bacteria (grey). (C) ROC curve with a 95% confidence interval (in brackets) generated from defined feature (ratio of integral of Raman shift: 1034.78/ 1061.79).

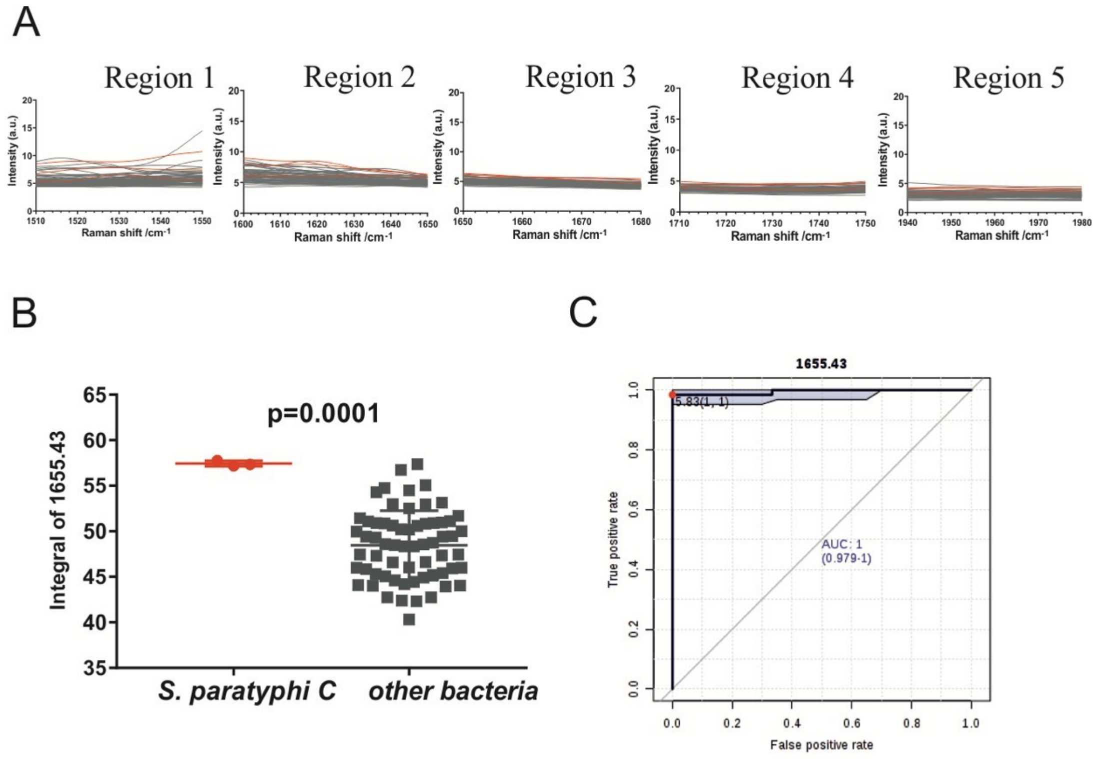

Figure S14. (A) SERS spectra of different region (selected) between the *S. paratyphi* C (red) and other 21 bacteria (grey). (B) Integral of Raman shift: 1655.43 in the *S. paratyphi* C (red) and other 21 bacteria (grey). (C) ROC curve with a 95% confidence interval (in brackets) generated from defined feature (integral of Raman shift: 1655.43).

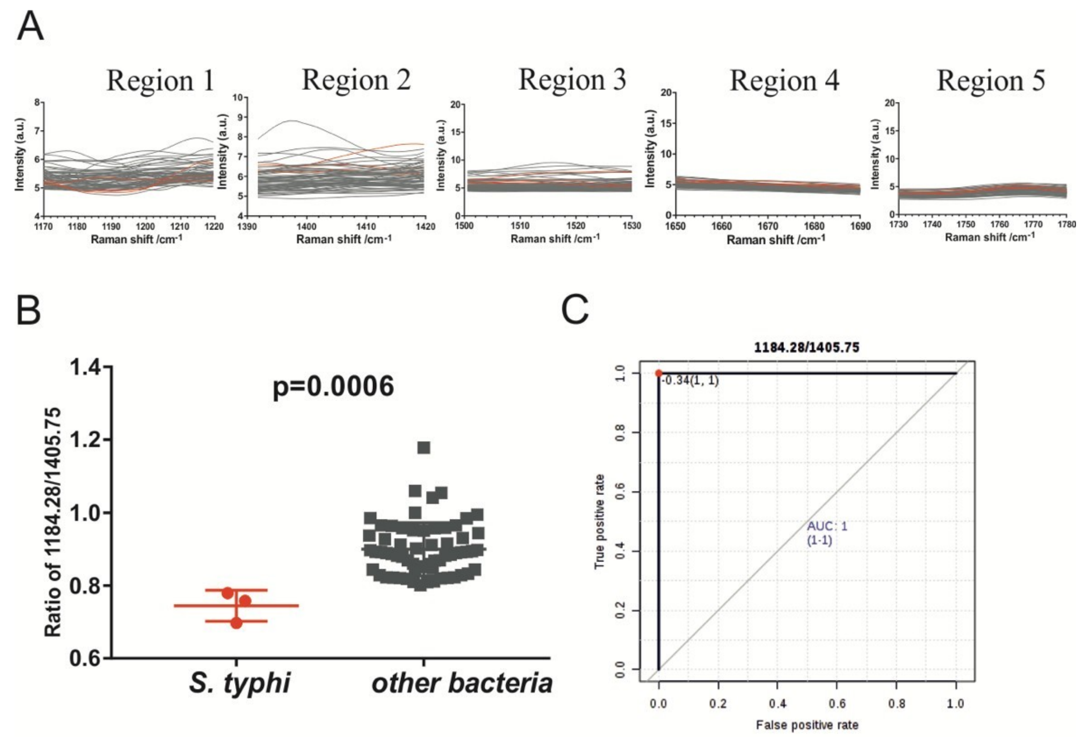

Figure S15. (A) SERS spectra of different region (selected) between the *S. typhi* (red) and other 21 bacteria (grey). (B) Ratio of integral of Raman shift: 1184.28/ 1405.75 in the *S. typhi* (red) and other 21 bacteria (grey). (C) ROC curve with a 95% confidence interval (in brackets) generated from defined feature (ratio of integral of Raman shift: 1184.28/ 1405.75).

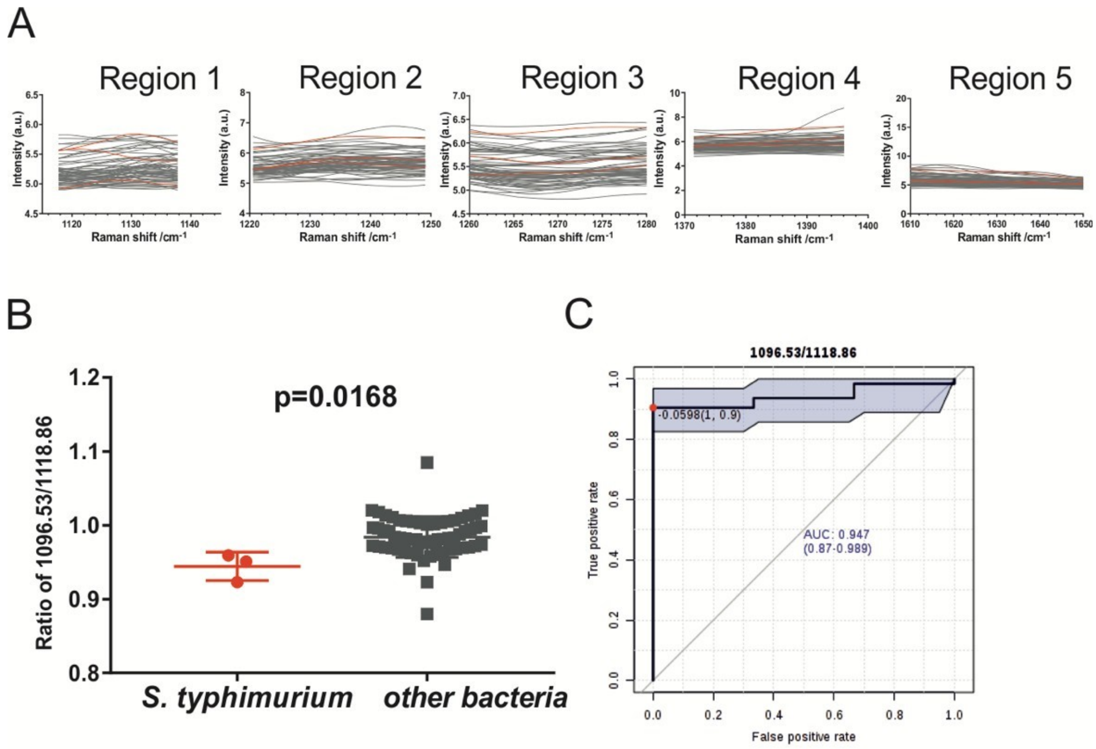

Figure S16. (A) SERS spectra of different region (selected) between the *S. typhimurium* (red) and other 21 bacteria (grey). (B) Ratio of integral of Raman shift: 1096.53/ 1118.86 in the *S. typhimurium* (red) and other 21 bacteria (grey). (C) ROC curve with a 95% confidence interval (in brackets) generated from defined feature (ratio of integral of Raman shift: 1096.53/ 1118.86).

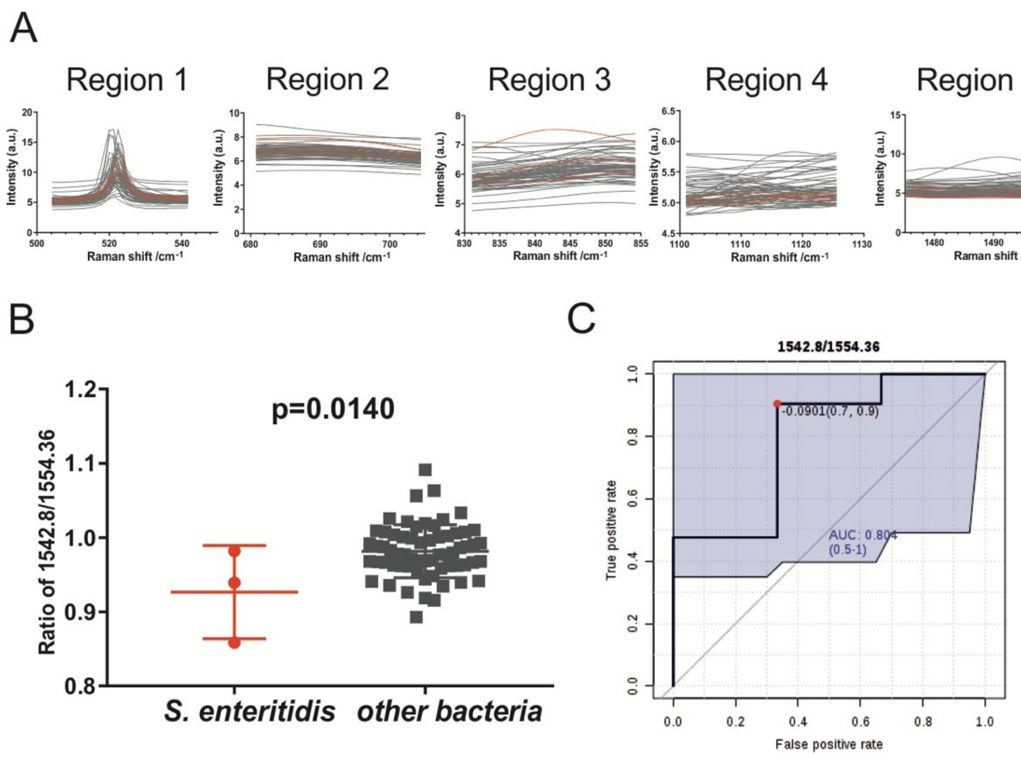

Figure S17. (A) SERS spectra of different region (selected) between the *S. enteritidis* (red) and other 21 bacteria (grey). (B) Ratio of integral of Raman shift: 1542.8/ 1554.36 in the *S. enteritidis* (red) and other 21 bacteria (grey). (C) ROC curve with a 95% confidence interval (in brackets) generated from defined feature (ratio of integral of Raman shift: 1542.8/ 1554.36).

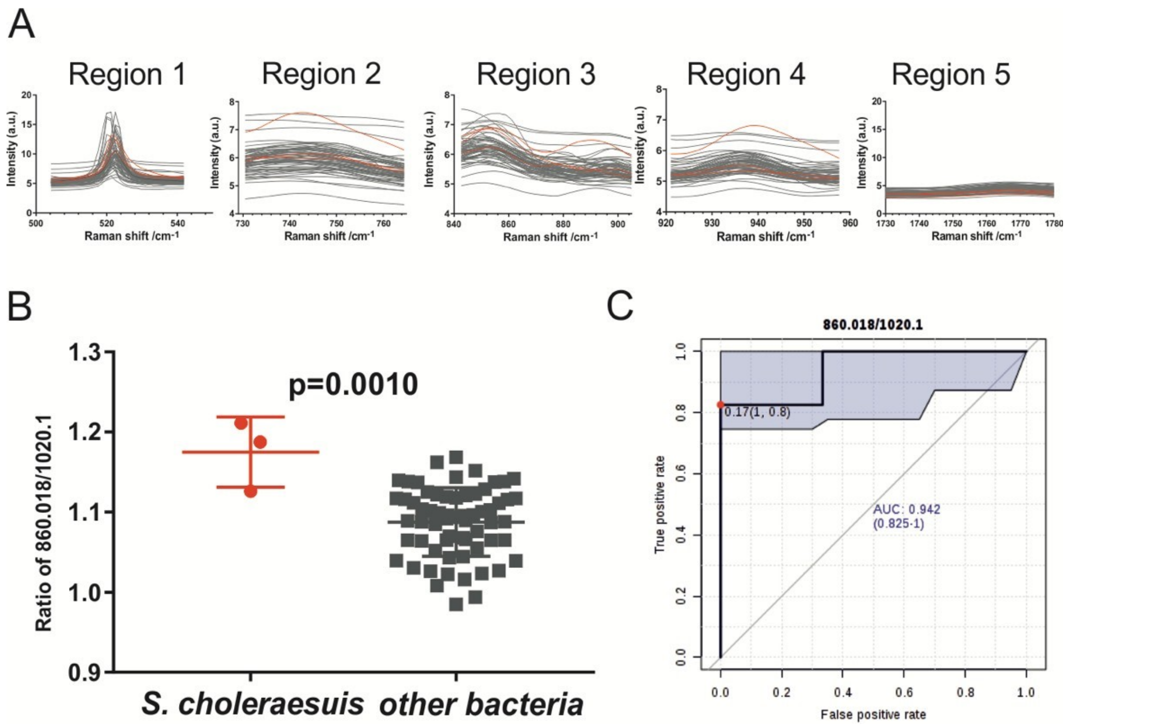

Figure S18. (A) SERS spectra of different region (selected) between the *S. choleraesuis* (red) and other 21 bacteria (grey). (B) Ratio of integral of Raman shift: 860.018/ 1020.1 in the *S. choleraesuis* (red) and other 21 bacteria (grey). (C) ROC curve with a 95% confidence interval (in brackets) generated from defined feature (ratio of integral of Raman shift: 860.018/ 1020.1).

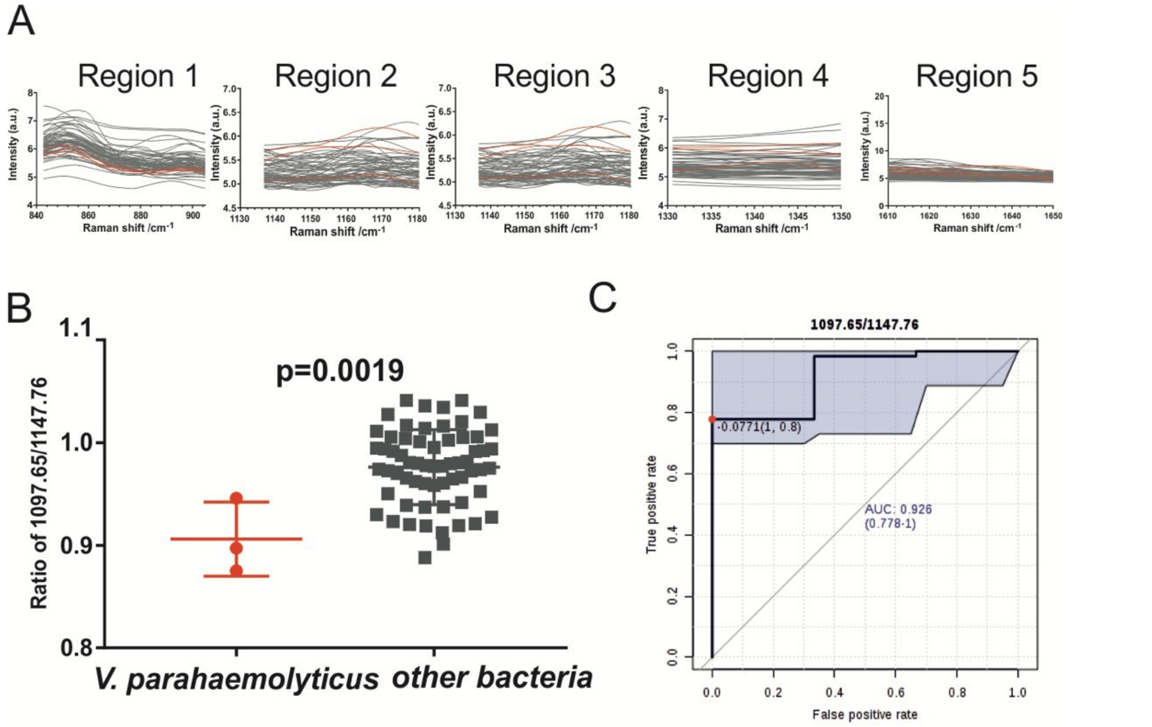

Figure S19. (A) SERS spectra of different region (selected) between the *V. parahaemolyticus* (red) and other 21 bacteria (grey). (B) Ratio of integral of Raman shift: 1097.65/ 1147.76 in the *V. parahaemolyticus* (red) and other 21 bacteria (grey). (C) ROC curve with a 95% confidence interval (in brackets) generated from defined feature (ratio of integral of Raman shift: 1097.65/ 1147.76).

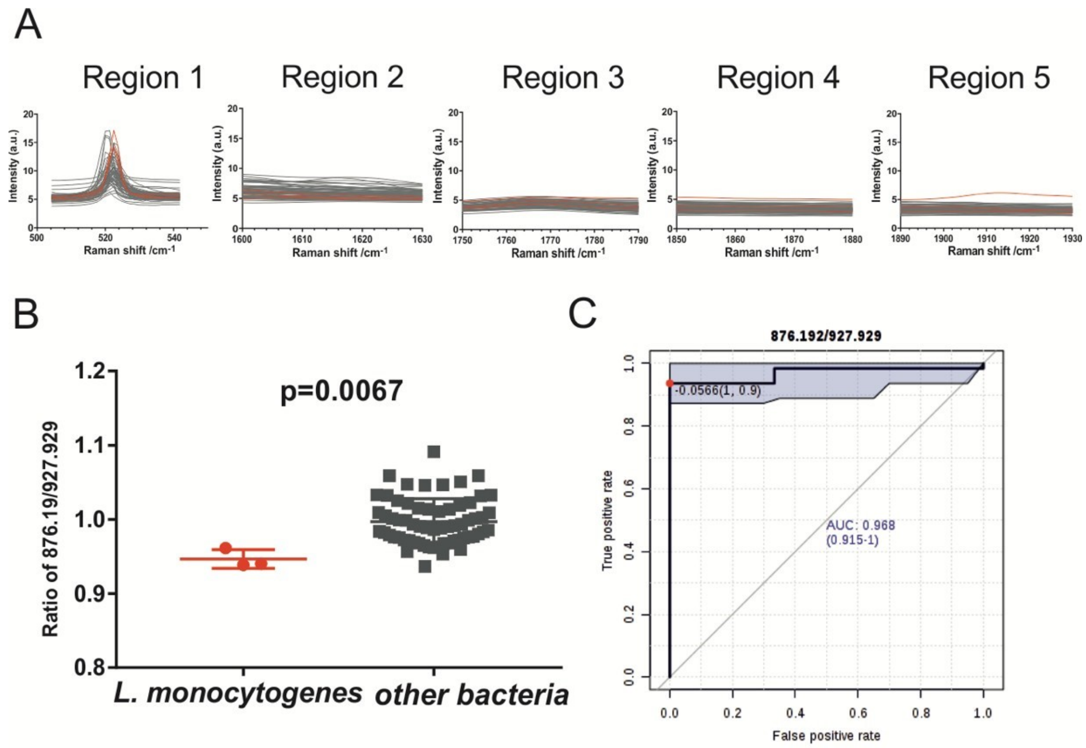

Figure S20. (A) SERS spectra of different region (selected) between the *L. monocytogenes* (red) and other 21 bacteria (grey). (B) Ratio of integral of Raman shift: 876.19/ 927.929 in the *L. monocytogenes* (red) and other 21 bacteria (grey). (C) ROC curve with a 95% confidence interval (in brackets) generated from defined feature (ratio of integral of Raman shift: 876.19/ 927.929).

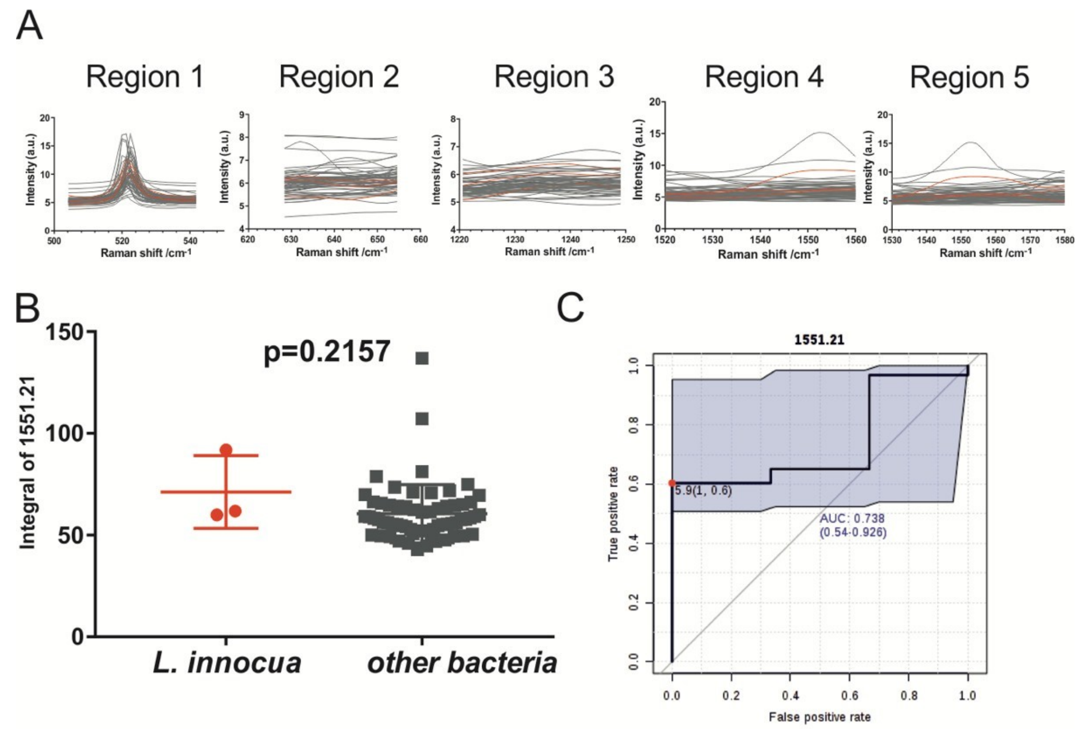

Figure S21. (A) SERS spectra of different region (selected) between the *L. innocua* (red) and other 21 bacteria (grey). (B) Integral of Raman shift: 1551.21 in the *L. innocua* (red) and other 21 bacteria (grey). (C) ROC curve with a 95% confidence interval (in brackets) generated from defined feature (integral of Raman shift: 1551.21).

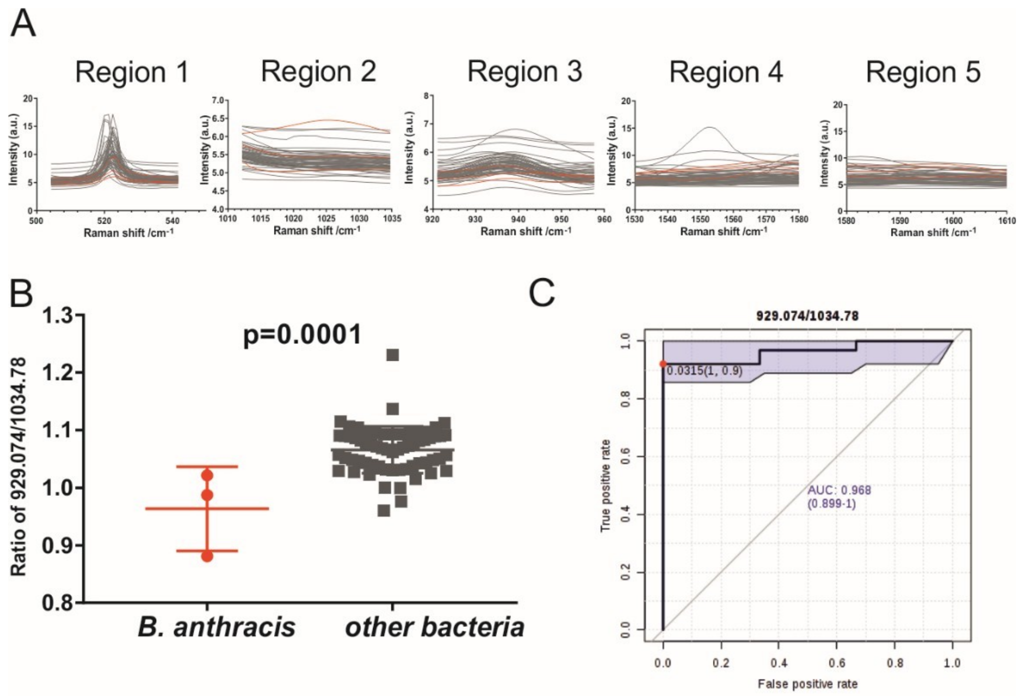

Figure S22. (A) SERS spectra of different region (selected) between the *B. anthracis* (red) and other 21 bacteria (grey). (B) Ratio of integral of Raman shift: 929.074/ 1034.78 in the *B. anthracis* (red) and other 21 bacteria (grey). (C) ROC curve with a 95% confidence interval (in brackets) generated from defined feature (ratio of integral of Raman shift: 929.074/ 1034.78).

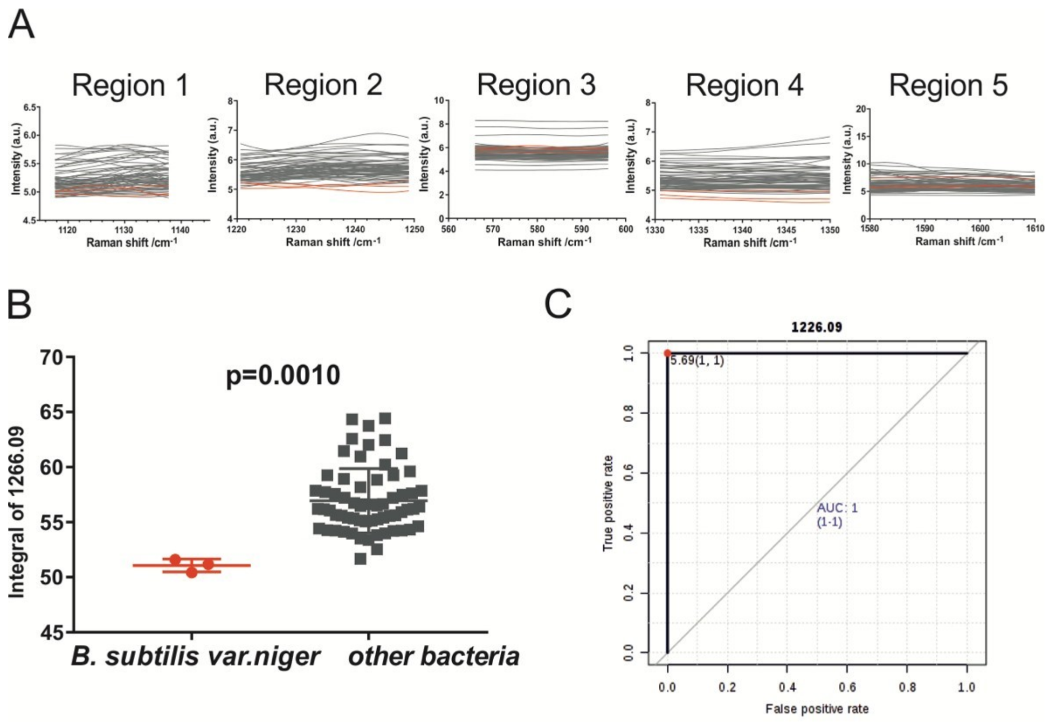

Figure S23. (A) SERS spectra of different region (selected) between the *B. subtilis var.niger* (red) and other 21 bacteria (grey). (B) Integral of Raman shift: 1266.09 in the *B. subtilis var.niger* (red) and other 21 bacteria (grey). (C) ROC curve with a 95% confidence interval (in brackets) generated from defined feature (integral of Raman shift: 1266.09).

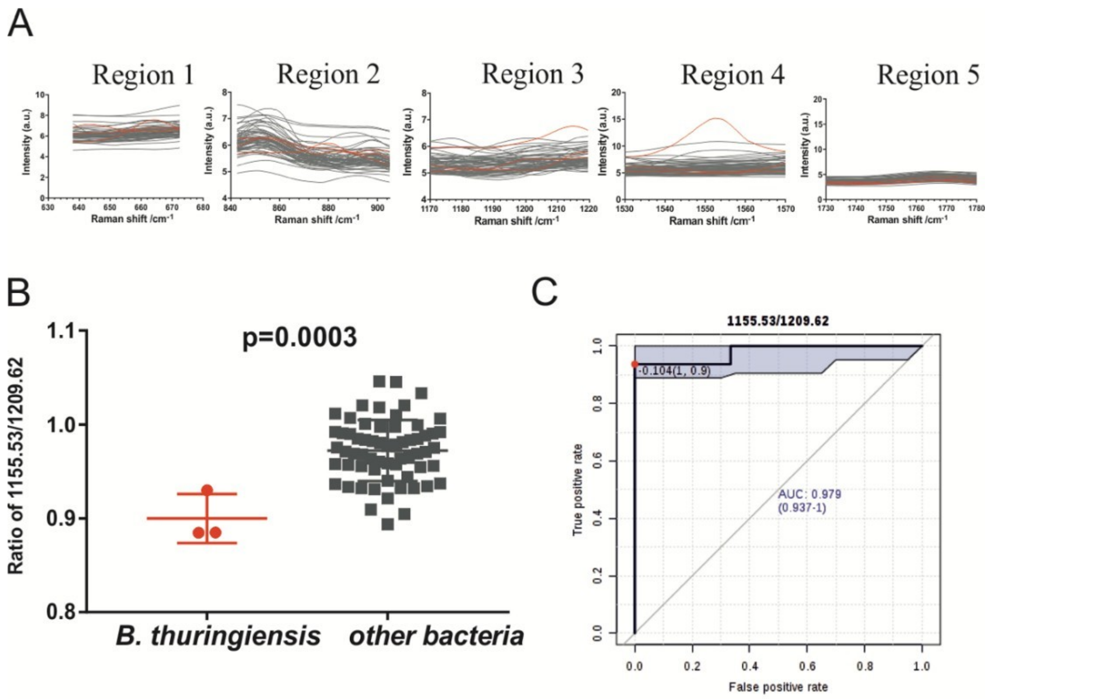

Figure S24. (A) SERS spectra of different region (selected) between the *B. thuringiensis* (red) and other 21 bacteria (grey). (B) Ratio of integral of Raman shift: 1155.53/ 1209.62 in the *B. thuringiensis* (red) and other 21 bacteria (grey). (C) ROC curve with a 95% confidence interval (in brackets) generated from defined feature (ratio of integral of Raman shift: 1155.53/ 1209.62).

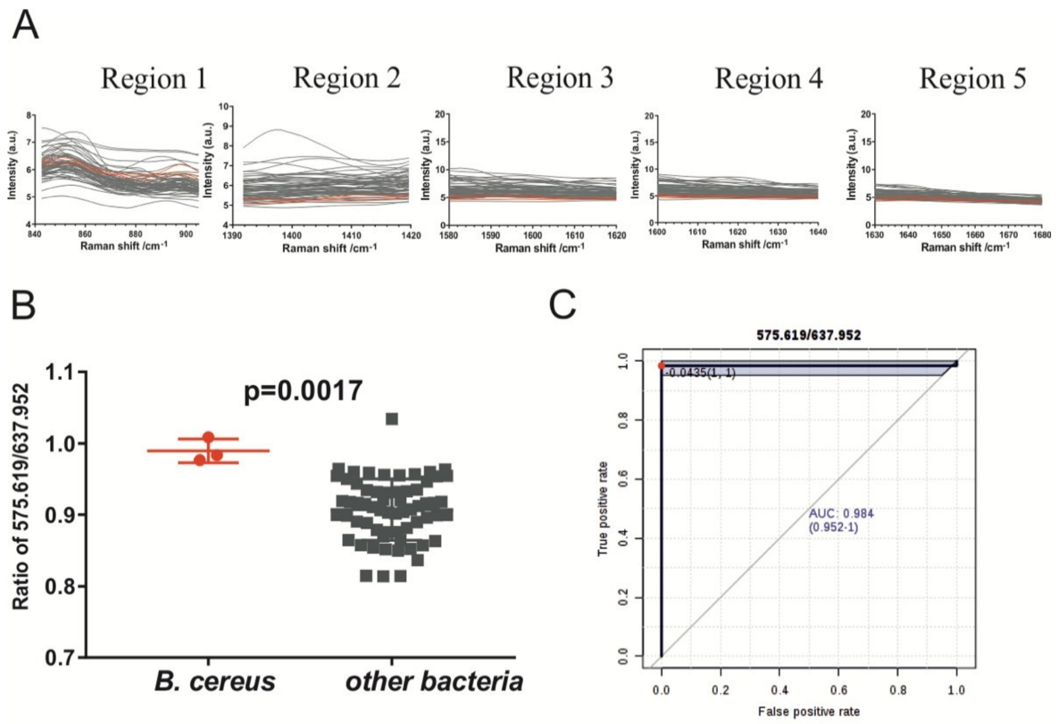

Figure S25. (A) SERS spectra of different region (selected) between the *B. cereus* (red) and other 21 bacteria (grey). (B) Ratio of integral of Raman shift: 575.619/ 637.952 in the *B. cereus* (red) and other 21 bacteria (grey). (C) ROC curve with a 95% confidence interval (in brackets) generated from defined feature (ratio of integral of Raman shift: 575.619/ 637.952).

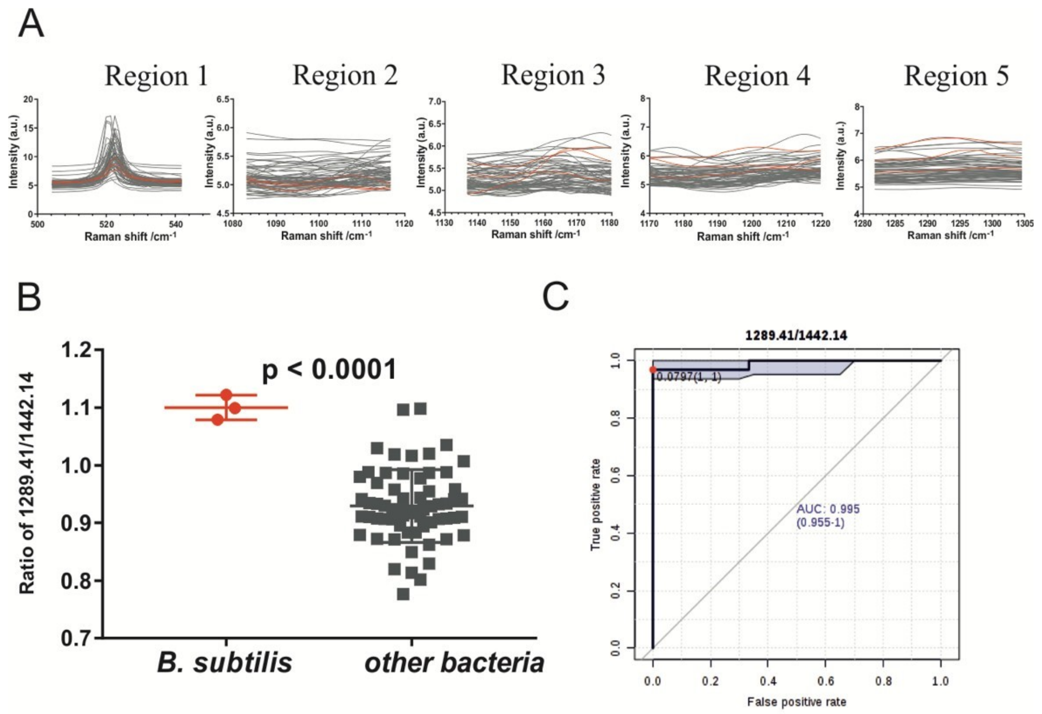

Figure S26. (A) SERS spectra of different region (selected) between the *B. subtilis* (red) and other 21 bacteria (grey). (B) Ratio of integral of Raman shift: 1289.41/ 1442.14 in the *B. subtilis* (red) and other 21 bacteria (grey). (C) ROC curve with a 95% confidence interval (in brackets) generated from defined feature (ratio of integral of Raman shift: 1289.41/ 1442.14).

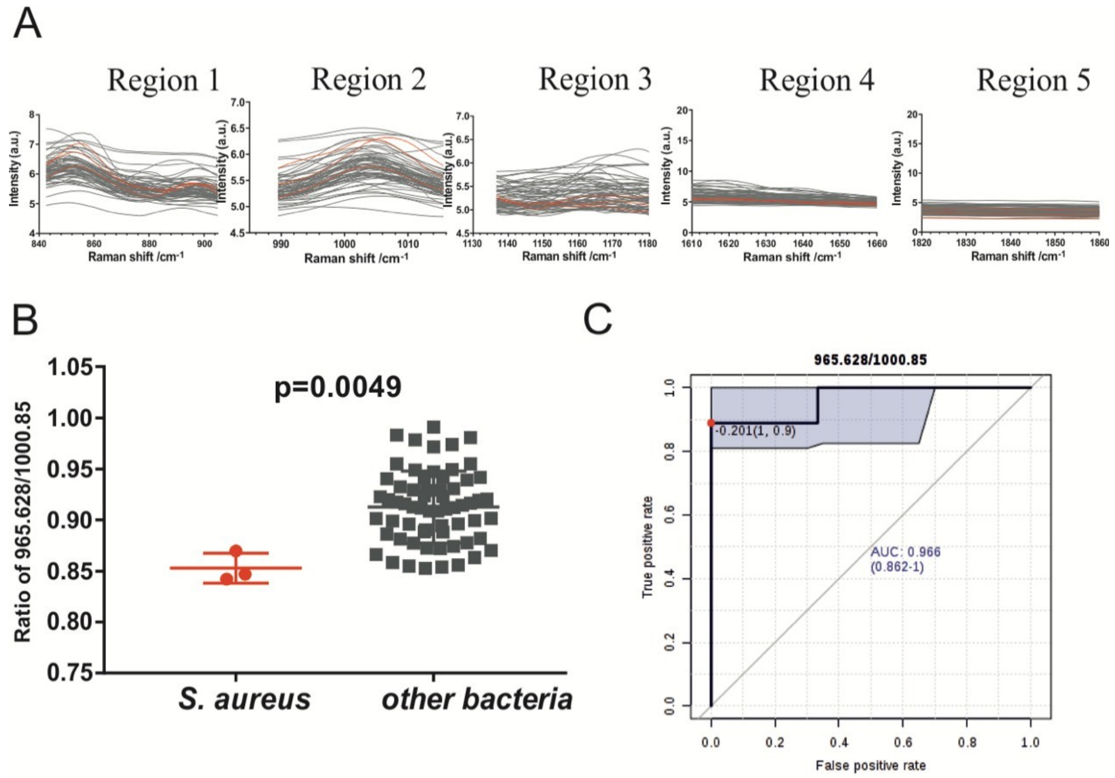

Figure S27. (A) SERS spectra of different region (selected) between the *S. aureus* (red) and other 21 bacteria (grey). (B) Ratio of integral of Raman shift: 965.628/ 1000.85 in the *S. aureus* (red) and other 21 bacteria (grey). (C) ROC curve with a 95% confidence interval (in brackets) generated from defined feature (ratio of integral of Raman shift: 965.628/ 1000.85).

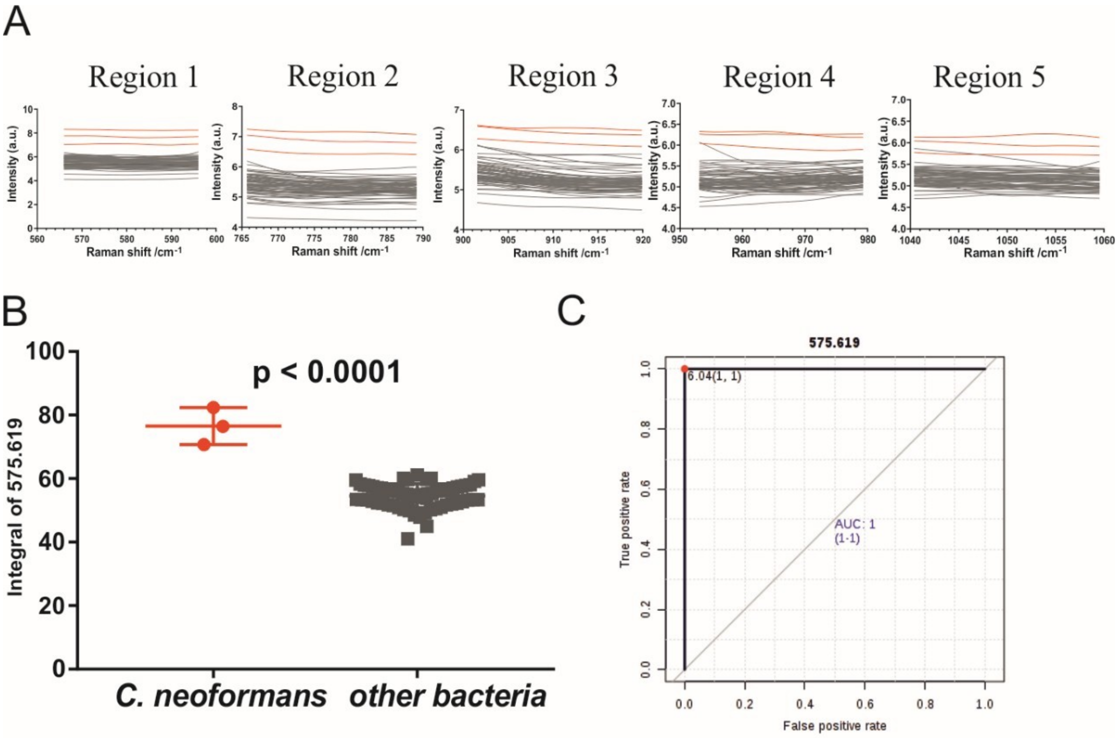

Figure S28. (A) SERS spectra of different region (selected) between the *C. neoformans* (red) and other 21 bacteria (grey). (B) Integral of Raman shift: 575.619 in the *C. neoformans* (red) and other 21 bacteria (grey). (C) ROC curve with a 95% confidence interval (in brackets) generated from defined feature (integral of Raman shift: 575.619).

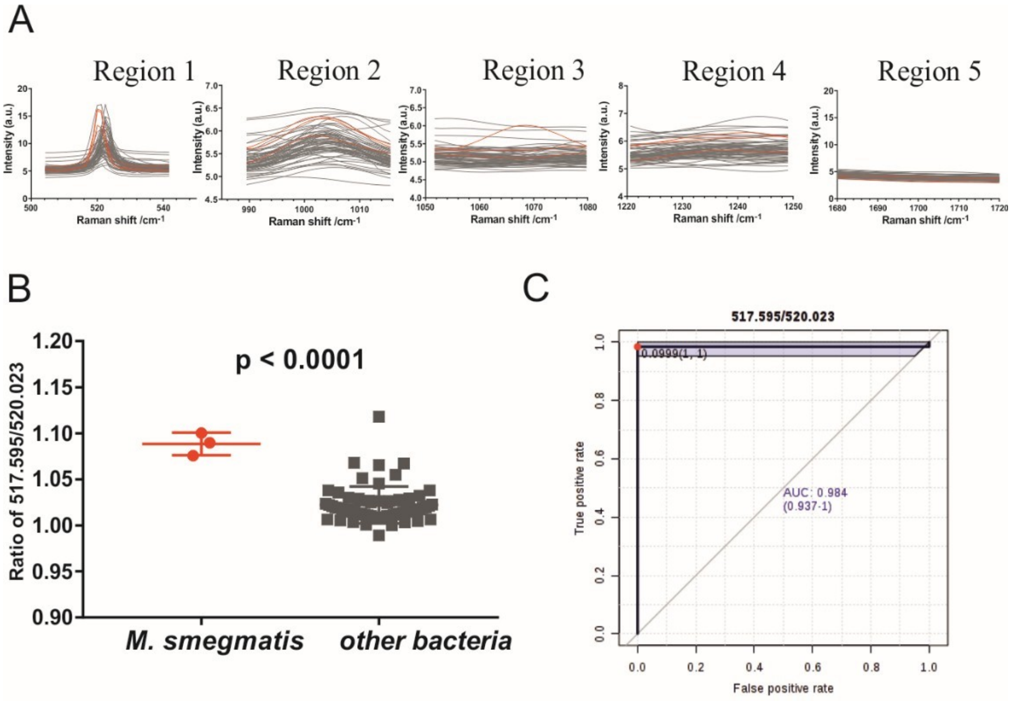

Figure S29. (A) SERS spectra of different region (selected) between the *M. smegmatis* (red) and other 21 bacteria (grey). (B) Ratio of integral of Raman shift: 517.595/ 520.023 in the *M. smegmatis* (red) and other 21 bacteria (grey). (C) ROC curve with a 95% confidence interval (in brackets) generated from defined feature (ratio of integral of Raman shift: 517.595/ 520.023).

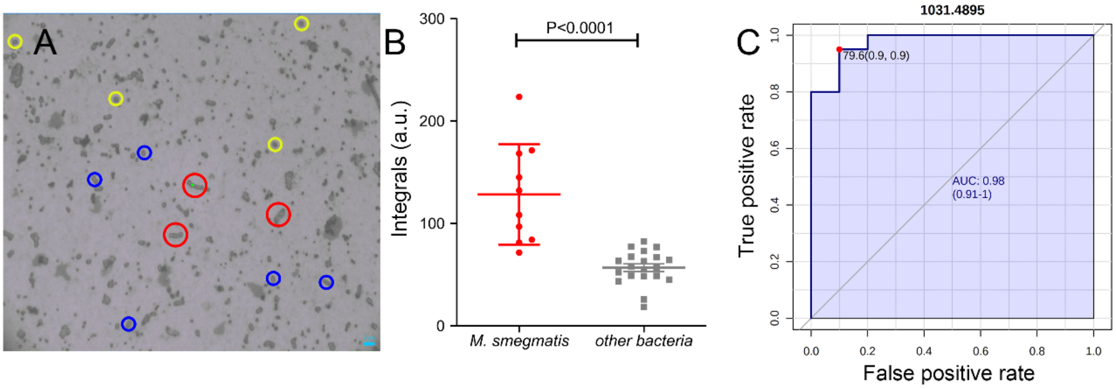

Figure S30. (A) Microscopic image of bacteria mixture (contains of *M. smegmatis* (Mycobacterium, marked in red circle), *S. aureus* (Coccus, marked in yellow circle), *E, coli O157*, *S. paratyphi* A, *V. parahaemolyticus* and *L. monocytogenes* (Bacterium, marked in blue circle)) in Raman system. (B) Integral of Raman shift: 1031 in the *M. smegmatis* (red) and other bacteria (grey). (C) ROC curve with a 95% confidence interval (in brackets) generated from defined feature (integral of Raman shift: 1031).

Table S1. The source of bacteria and culture medium. BNCC: BeNa Culture Collection

Source	bacteria	culture medium
Anti-plague Institute Hebei Province	B. melitensis	Luria-Bertani
Beijing Institute of Microbiology and Epidemiology	F. tularensis	Alkaline Peptone Broth
Anti-plague Institute Hebei Province	Y. pestis	Luria-Bertani
Beijing Institute of Microbiology and Epidemiology	E. coli O157	Luria-Bertani
BNCC337823	S. paratyphi A	Luria-Bertani
BNCC103389	S. paratyphi B	Luria-Bertani
BNCC339948	S. paratyphi C	Luria-Bertani
BNCC108331	S. typhi	Luria-Bertani
BNCC108207	S. typhimurium	Luria-Bertani
BNCC353759	S. enteritidis	Luria-Bertani
BNCC126547	S. choleraesuis	Luria-Bertani
BNCC105263	V. parahaemolyticus	Luria-Bertani
BNCC338021	L. monocytogenes	Brain Heart Infusion Agar
BNCC139467	L. innocua	Luria-Bertani
Beijing Institute of Microbiology and Epidemiology	B. anthracis	Luria-Bertani
Beijing Institute of Microbiology and Epidemiology	B. subtilis var.niger	Luria-Bertani

BNCC136456	B. thuringiensis	Luria-Bertani
BNCC103930	B. cereus	Luria-Bertani
BNCC188062	B. subtilis	Luria-Bertani
BNCC310011	S. aureus	Luria-Bertani
BNCC337347	C. neoformans	YM Medium
BNCC104116	M. smegmatis	Brain Heart Infusion Agar

Table S2. Area under the ROC curve (AUC) values obtained from ROC analysis for the detection of all bacteria.

classification: Classification of dyeing	Morphological classification	species	bacteria	Region 1	Region 2	Region 3	Region 4	Region 5	AUC	confidence interval	p-value
G-	Bacterium	brucella	*B. melitensis*	518	1000	1101	1208	1761	0.926	0.783-1.0	0.0053
		francisella	*F. tularensis*	523	886	1495	1509	1622	0.947	0.857-1.0	0.0092
		yersinia	*Y. pestis*	971	1092	1099	1584	1613	0.974	0.915-1.0	0.0039
		escherichia	*E, coli* O157	518	888	1167	1190	1799	0.974	0.921-1.0	<0.0001
		salmonella	*S. paratyphi* A	1177	1446	1461	1485	1518	1	1.0	<0.0001
			S. paratyphi B	752	868	939	1007	1039	0.926	0.794-1.0	0.0090
			S. paratyphi C	1530	1625	1668	1731	1964	1	0.979-1.0	0.0001
			S. typhi	1189	1410	1511	1673	1759	1	1.0	0.0006
			S. typhimurium	1122	1231	1270	1385	1634	0.947	0.87-0.989	0.0168
			S. enteritidis	524	692	841	1108	1496	0.804	0.5-1.0	0.0140
			S. choleraesuis	526	742	855	945	1759	0.942	0.825-1.0	0.0010
	Vibrio	Vibrio	*V. parahaemolyticus*	863	1154	1165	1339	1636	0.926	0.778-1.0	0.0019
G+	Bacterium	listeria	*L. monocytogenes*	522	1617	1774	1869	1910	0.968	0.915-1.0	0.0067
			L. innocua	521	642	1237	1545	1559	0.738	0.54-0.926	0.2157
		bacillus	*B. anthracis*	521	1024	932	1566	1596	0.968	0.899-1.0	0.0001
			B. subtilis var.niger	1132	1234	1313	1340	1598	1	1.0	0.0010
			B. thuringiensis	660	878	1214	1551	1755	0.979	0.937-1.0	0.003
			B. cereus	898	1402	1605	1621	1657	0.984	0.952-1.0	0.0017
			B. subtilis	521	1103	1167	1197	1294	0.995	0.955-1.0	<0.0001
	Coccus	staphylococcus	*S. aureus*	854	1006	1145	1638	1842	0.966	0.862-1.0	0.0049
			C. neoformans	580	777	911	963	1053	1	1.0	<0.0001
acid-fast staining	Mycobacterium	mycobacteriu	*M. smegmatis*	521	999	1066	1233	1701	0.984	0.937-1.0	<0.0001

Table S3. Major spectral band assignments for bacteria.[1-3]

Raman Shift /cm^{-1}	Band assignments
~520	Si wafer
580	C-O-C glycosidic ring deformation
640-660	Nucleic acids
742-777	Adenine, FAD, NAG
841	Cytosine, uracil, tyrosine
~855	ring breathing Tyr protein
911-932	C-C stretching modes in proteins
939-971	C-N stretching modes
~1000	Phenylalanine ring breathing
1024-1066	C-C ring breathing
~1100	C-O stretching (amides), proteins
1122	NH^{3+} formation vibration
1130-1180	Amide, DNA, C-N stretching vibration of adenine
1216-1295	Amide III
1313-1340	Adenine breathing ring
1385	Thymine breathing ring
1498-1410	C-O vibration, COO- symmetrical vibration
1430-1461	CH_2 deformation lipids, proteins, carbohydrates
1494-1509	Phe
1516-1553	C-H bend or C=C stretching
1559-1605	C-N stretching proteins, DNA, Amides
1617-1621	C=C stretching olefinic

REFERENCES

(1) Liu, Y.; Zhou, H.; Hu, Z.; Yu, G.; Yang, D.; Zhao, J. Label and label-free based surface-enhanced Raman scattering for pathogen bacteria detection: A review. *Biosens. Bioelectron* **2017**, *94*, 131-140.

(2) Alula, M. T.; Krishnan, S.; Hendricks, N. R.; Karamchand, L.; Blackburn, J. M. Identification and quantitation of pathogenic bacteria via in-situ formation of silver nanoparticles on cell walls, and their detection via SERS. *Microchim. Acta* **2016**, *184*, 219-227.

(3) Chisanga, M.; Muhamadali, H.; Ellis, D. I.; Goodacre, R. Surface-Enhanced Raman Scattering (SERS) in Microbiology: Illumination and Enhancement of the Microbial World. *Appl. Spectrosc.* **2018**, *72*, 987-1000.
